# Uncovering Structure–Conductivity Relationships in Anion Exchange Membranes (AEMs) Using Interpretable Machine Learning

**DOI:** 10.3390/membranes16010012

**Published:** 2025-12-31

**Authors:** Pegah Naghshnejad, Debojyoti Das, Jose A. Romagnoli, Revati Kumar, Jianhua Chen

**Affiliations:** 1 Department of Chemical Engineering, Louisiana State University, Baton Rouge, LA 70803, USA; pnaghs1@lsu.edu; 2 Department of Chemistry, Louisiana State University, Baton Rouge, LA 70803, USA; ddas7@lsu.edu (D.D.); revatik@lsu.edu (R.K.); 3 Department of Computer Science, Louisiana State University, Baton Rouge, LA 70803, USA; cschen@lsu.edu

**Keywords:** anion exchange membranes, anion conductivity, machine learning, data driven modeling

## Abstract

Anion exchange membranes (AEMs) play a vital role in the performance of water electrolyzers and fuel cells, yet their discovery and optimization remain challenging due to the complexity of structure–property relationships. In this study, we introduce a machine learning framework that leverages conditional graph neural networks (cGNNs) and descriptor-based models and a hybrid graph neural network (HGARE) to predict and interpret ionic conductivity. The descriptor-based pipeline employs principal component analysis (PCA), ablation, and SHAP analysis to identify factors governing anion conductivity, revealing electronic, topological, and compositional descriptors as key contributors. Beyond prediction, dimensionality reduction and clustering are performed by employing t-SNE and KMeans as well as SOM, which reveal distinct membranes clusters, some of which were enriched with high anion conductivity. Among graph-based approaches, the graph convolutional (GCN) achieved strong predictive performance, while the Hybrid Graph Autoencoder-Regressor Ensemble (HGARE) achieved the highest accuracy. Additionally, atom-level saliency maps from GCN provide spatial explanations for conductive behavior, revealing the importance of polarizable and flexible regions. This work contributes to the accelerated and data-driven design of high-performance AEMs.

## 1. Introduction

Anion exchange membranes (AEMs) play a crucial role in electrochemical energy systems, such as alkaline fuel cells and water electrolyzers, and facilitate the selective transport of hydroxide ions (${OH}^{-}$). Their development offers significant advantages over proton exchange membranes by enabling the use of non-noble metal catalysts and cost-effective cell components [1]. However, limitations such as lower hydroxide ion mobility, instability of cationic groups in alkaline media, and insufficient mechanical durability pose ongoing challenges [2,3,4].

Traditionally, progress in the development of anion exchange membranes for electrochemical energy conversion and separation technologies has been achieved primarily through trial-and-error experimental approaches. In these workflows, new polymer backbones, side chains, or cationic headgroups are systematically synthesized and subsequently evaluated for the electrochemical performance. Such workflows are time-consuming and inefficient, given the vast design space of potential polymers. Notably, minor changes in molecular architecture can influence ion conductivity and membrane swelling [3]. The identification of informative molecular features is critical for predictive accuracy [5]. The Mordred package generates thousands of physicochemical, topological, and electronic features that capture subtle structural variations [6]. To address these challenges, machine learning (ML) has emerged as a powerful approach for predicting key polymer properties and guiding material discovery. Recent developments demonstrate how deep learning models are successfully applied across domains, ranging from polymer chemistry to medical diagnostics [7,8,9].

ML and data-driven modeling are increasingly applied in material science [10,11,12], enabling the accelerated screening and prediction of polymer properties. Data-driven models can extract complex structure–property relationships from high-dimensional molecular descriptors and enable efficient design of high-performance AEMs [1,2,13]. ML models can learn complex structure–property relationships from existing data, enabling researchers to screen and optimize new AEM candidates virtually before synthesis [14]. Zhai et al. developed a deep learning model to predict ${OH}^{-}$ conductivity in poly (2,6)-dimethyl phenylene oxide-based AEMs, achieving high accuracy across different functional group chemistries [15]. Similarly, Phua et al. applied explainable ML to jointly predict conductivity and alkaline stability, identifying compositional and topological features that distinguish high-performance materials [16]. In related work, MOF-based membranes with tailored/hydrophilic/hydrophobic balance and improved mechanical robustness demonstrate the significance of combining stability with next-generation AEM design [17]. For further discussion of topological design in materials, see Deng et al. [18].

This field is also moving towards sustainability-driven polymer design. Most commercial AEMs rely on fluorinated backbones or unstable cationic groups. To overcome these issues, Schertzer et al. employed ML to design fluorine-free AEMs that balance high ${OH}^{-}$ conductivity with low swelling and high alkaline stability. Their virtual screening campaign identified over 400 high-performing, fluorine-free candidates from millions of hypothetical polymers [19]. These align with the recommendations from Yassin et al. who emphasize that future AEMs must achieve a balance between conductivity, stability, and durability to meet fuel cell operation targets [2]. Together, these works illustrate the growing convergence of polymer chemistry, data science, and explainable ML.

Graph neural networks (GNNs) such as graph convolutional networks (GCNs) [20,21,22], have shown promise in polymer informatics due to their ability to process molecular graphs rather than precomputed descriptors [23,24]. This capability is rooted in advances in message-passing neural networks that directly learn atom-level features from graph topology, which have been widely applied in molecular property prediction [25,26]. This allows models to directly learn from atomic and bonding information in the polymer backbone. Liu et al. used augmented GCN models to screen copolymers for AEM applications, ultimately guiding the synthesis of membranes that achieved high conductivity and favorable water uptake [27].

Despite the success of ML in property prediction, interpretability remains a key challenge. SHAP quantifies the contribution of each input feature to a given prediction, enabling the identification of key descriptors associated with high or low conductivity [28]. In a recent study, Dalal et al. used SHAP and Bayesian optimization to guide the design of gene delivery polymers, showing how model interpretations can uncover actionable chemical insights [29]. In the context of AEMs, SHAP analysis by Phua et al. revealed that some features were strongly associated with enhanced ion transport.

In addition to molecular descriptors and graph representations, we also considered several system-level design parameters that strongly influence anion conductivity but are not directly encoded in SMILES or molecular graphs. Specifically, the Block A fraction represents the hydrophilic, ion-conducting segment that enhances hydroxide ion transport through water uptake, while the Block B (same here as well) fraction corresponds to the hydrophobic, mechanically stabilizing domain that improves structural robustness and mitigates excessive swelling. Their ratio (Block A/B) is a critical trade-off parameter: higher Block A content facilitates conductivity but risks mechanical instability, whereas higher block B content improves durability but restricts ion mobility. We further distinguished between polymer type (copolymer vs. homopolymer). Finally, all conductivity measurements were at 80 °C, widely accepted benchmarking conditions that isolate structure–property relationships and eliminate temperature variability in ion transport.

We further employed saliency mapping [30,31] on GNNs to directly visualize which atoms and substructures more strongly contribute to predicted hydroxide conductivity. Unsupervised learning techniques are increasingly applied in polymer science to explore chemical space and uncover latent structural patterns. Dimensionality reduction algorithms such as t-SNE [32] and UMAP [33], when combined with clustering algorithms like DBSCAN [34], can reveal subpopulations of structurally or functionally similar membranes. Phua et al. used dimensionality reduction and clustering techniques to map AEMS structures into distinct clusters and showed that specific clusters were enriched in high-conductivity polymers [35]. Similarly, Ehiro et al. developed a method to interpret clusters using feature importance analysis, linking each chemical space region to dominant molecular characteristics [36].

Despite recent progress, a unified framework that integrates descriptor-based interpretability with graph-based generalization and unsupervised pattern discovery for AEM remains underexplored. Building on these foundations, this study introduces a multi-scale interpretable ML framework for predicting and explaining anion conductivity in AEMs. Our approach integrates descriptor-based modeling (using PCA, SHAP, ablation analysis) with graph-based learning (GCN, GAT) and a novel Hybrid Graph Autoencoder-Regressor Ensemble (HGARE). The framework not only enhances predictive accuracy but also provides chemical interpretability through feature attribution and saliency visualization. Furthermore, unsupervised models, t-SNE, KMeans, and SOM uncovers latent structural families associated with high anion conductivity. Together, these components bridge molecular-level descriptors and graph-level learning, accelerating the data-driven discovery of high-performance AEMs.

## 2. Methodology

**Figure 1:**
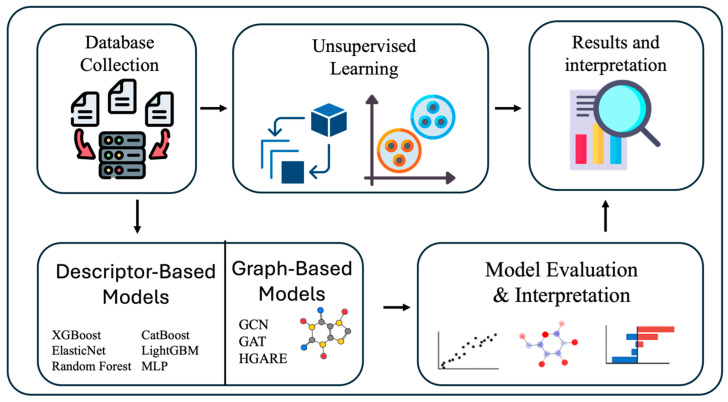
Workflow of proposed machine learning framework for predicting and interpreting anion conductivity of AEMs.

Our proposed ML framework for predicting and interpreting anion conductivity of AEMs is illustrated in Figure 1. Molecular descriptors were computed using Mordred [6].

For unsupervised analysis, t-SNE is combined with KMeans for data clustering. Self-Organizing Map (SOM) plots were used to identify descriptor trends within each cluster [10,37].

For supervised modeling, we trained two separate pipelines. The first model is based on deep learning, consisting of a GCN with two layers and ReLU activation, followed by a multi-layer perceptron (MLP) head [38]. In addition, we implemented a Graph Attention mechanism to weigh the importance of neighboring nodes, providing more expressive graph representations. Also, we used a HGARE model that combines graph autoencoder pretraining and supervised fine-tuning and ensemble averaging for enhanced prediction accuracy. The other model is a group of traditional ML regressors (including XGBoost, CatBoost, Random Forest, LightGBM, ElasticNet, MLP) trained on precomputed descriptors. The models were evaluated using $R^{2}$, MAE, and RMSE.

Descriptor-based models are advantageous because they are computationally efficient, relatively easy to train, and highly interpretable due to their reliance on chemically meaningful molecular descriptors. However, their performance is constrained by the expressiveness of predefined descriptors, which may not fully capture complex topological or electronic features present in AEM architectures.

Graph-based neural networks (GNNs) automatically learn hierarchical representations directly from molecular graphs, enabling richer characterization of polymer backbones, cross-linking patterns, and functional group connectivity. Their main limitations include higher data requirements and reduced interpretability relative to descriptor-based models.

Unsupervised learning methods (PCA, t-SNE, SOM, KMeans) serve as exploratory tools that reveal intrinsic structures within the descriptor space, helping identify regions of high-performance materials and trends in molecular diversity. These methods do not perform prediction directly but support model interpretation and design rule formulation.

### 2.1. Unsupervised Learning

Unsupervised learning or knowledge discovery is a machine learning area in which algorithms extract features and identify unlabeled dataset patterns. By leveraging unsupervised learning, researchers can uncover hidden structures and gain a more comprehensive understanding of the data [39,40].

After preprocessing, dimensionality reduction (DR) techniques project the process data, remove redundant, correlated data, and combine them into lower dimensional scores. DR may project data in two or three dimensions to enable visualization, or the technique may simply function to remove redundant information from process data. In this work, t-SNE was employed for dimensionality reduction with a perplexity of 30 and was chosen to balance local neighborhood preservation with global structure, the learning rate was set to 200 to avoid crowding effects, and the algorithm was run for 1000 iterations to ensure convergence.

After DR, clustering algorithms were applied to group the data into meaningful clusters without prior labels. The goal of data clustering is the unsupervised classification of data into groups or clusters that are useful and meaningful. Using data clustering, the key groups within a database can be isolated and connected to meaningful classification. In this work, a density-based clustering tool was considered. Here, we used KMeans [41] specifying three clusters. These values were empirically determined to yield the most stable and chemically meaningful separation of polymers. In this clustering method, regions of high density of any shape are located and separated from one another by regions of low density.

Once clusters are identified, verification of their separation is crucial before using supervised learning. To verify the separation between different clusters and provide a meaningful explanation, alternative methods such as Subspace Greedy Search (SGS) or SHAP analysis can be used to find the highest contributing combination of variables (descriptors in our application) that cause separation. A complete description of all these methods, hyperparameter tuning, and it applications to chemical processes is provided by Romagnoli et al. [42].

In the predictive phase, PCA was utilized to construct regression models. PCA compressed the original 1432 Mordred descriptors into eight orthogonal principal components, retaining 80.54% of the total variance, effectively minimizing collinearity and overfitting in the regression stage. These principal components were subsequently used as inputs for the descriptor-based models which achieved high R^2^ values (0.85), demonstrating that PCA-based latent features successfully preserved the essential structural–electronic information required for accurate anion conductivity prediction.

### 2.2. Descriptor-Based Model

**Figure 2:**
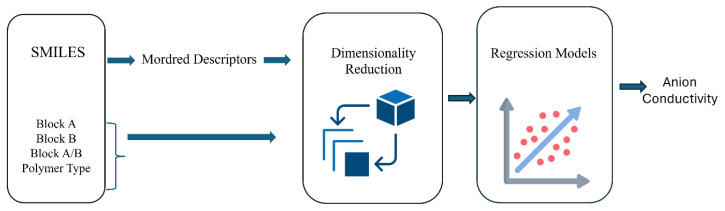
Schematic of descriptor-based prediction pipeline.

To develop a robust descriptor-based model for predicting the ionic conductivity of AEMs, we utilized Mordred, a widely used molecular descriptor calculator that supports over 1800 two-dimensional and three-dimensional descriptors [6]. Each polymer backbone or repeat unit is represented as a SMILES string, and then the SMILES strings are converted to Mordred descriptors. Mordred is an open-source molecular descriptor calculator [6] capable of rapidly computing an extensive set of features of each molecule. However, certain descriptors may be undefined for specific molecules, resulting in missing values. To ensure data quality, we applied strict data cleaning. We removed descriptors containing missing values across polymers, yielding a high-quality dataset with 207 AEMs and 1432 numeric descriptors, each free of missing or invalid values. Additionally, composition-based features were included to reflect polymer microstructure, alongside categorical information such as polymer type. These descriptors were subsequently used as input features for training supervised machine learning models to predict the membranes of anion conductivity (Figure 2).

To ensure robust generalization and minimize overfitting due to high dimensionality, PCA [43] was applied prior to the model training, reducing the descriptor space to eight principal components explaining 80.54 of the total variances. The resulting features were then used to train and evaluate multiple regression algorithms, including CatBoost [44], XGBoost [45], Random Forest [46], LightGBM [47], ElasticNet [48], and multi-layer Perceptron (MLP) neural network [49].

### 2.3. Graph Models

In the graph-based models, each polymer was represented as a molecular graph, where each atom served as nodes and bonds as edges. Each atom in the polymer is represented by a 74-dimensional feature vector. This vector encodes basic chemical properties such as atom type, degree (number of bonded neighbors), formal charge, aromaticity, and hybridization state (e.g., sp, sp^2^, sp^3^). Additional flags mark whether the atom belongs to a ring and their categorical descriptors generated with RDKIT. These atom-level features serve as the input to the GNN layers. Graph-level embeddings were obtained through two stacked convolutional layers, followed by mean pooling to aggregate node representation into a single molecular vector. Mean pooling was selected instead of sum or max pooling because it normalizes variations in molecular, size, ensuring comparability across polymers with different chain lengths.

#### 2.3.1. Graph Convolutional Network

In this model, we used two layers of graph convolution operations [38], implemented using the DGL library. Each layer applies the following Transformation:

(1) $$h_{v}^{\left(l+1\right)}=ReLU(\sum_{u\in N\left(V\right)}W^{\left(l\right)}h_{u}^{\left(l\right)}+b^{\left(l\right)}),$$

where N(*v*) denotes the neighborhood of node *v*, and $W^{\left(l\right)},b^{\left(l\right)}$ are trainable parameters of the *l*-th GCN layer. After two such layers, node embeddings are aggregated using mean pooling to obtain graph-level representation $h_{G}$.

The representation is concatenated with the input molecular descriptors and passed through a three-layer MLP with ReLU activation to predict the target property. Formally,

(2) $$Y^{\wedge }=f_{MLP}\left(\mathrm{Concat}\;(h_{G},features)\right)$$

where *features* represent other molecular features.

**Figure 3:**
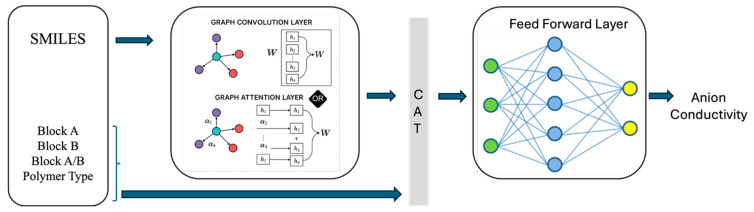
Architecture of the hybrid GCN model.

Figure 3 illustrates the overall architecture of our graph-based model. In this hybrid framework, molecular structure is encoded via a graph convolutional layer that learns atom-level embeddings through neighborhood aggregation. These node embeddings are pooled into a graph-level vector and concatenated with hand-crafted molecular descriptors and polymer composition features, including the percentage of Block A, percentage of Block B, and the block A/B ratio.

Block A and Block B correspond to the two polymer segments or building blocks within the copolymer structure. The Block A fraction represents the hydrophilic or ion-conducting segment, which facilitates hydroxide ion transport, while Block B represents the hydrophobic or mechanically stable segment that enhances structural integrity. The Block A/B ratio captures the relative proportion of these segments and reflects the balance between ion conductivity and mechanical durability.

The combined representation is passed through a feed-forward layer to predict anion conductivity. This design enables the model to simultaneously leverage structural information from the molecular graph and composition features.

#### 2.3.2. Graph Attention Network

To improve expressive power, the GAT model replaces standard graph convolutions with Graph Attention layers, enabling the network to learn dynamic, edge-aware attention weights across neighbors. Each attention layer computes the following:

(3) $$h_{v}^{\left(l+1\right)}={∥}_{k=1}^{K}ReLU(\sum_{u\in N\left(v\right)}{\alpha_{vu}^{\left(k\right)}W}^{\left(k\right)}+h_{u}^{\left(l\right)}),$$

where $\alpha_{vu}^{\left(k\right)}$ are the attention coefficients learned from the $k^{th}$ attention head, $W^{\left(k\right)}$ are the corresponding projection weights, and *K* is the number of heads. Two stacked GAT layers are applied, followed by mean node pooling and concatenation as in the GCN. The combined feature vector is passed through an MLP to produce the final prediction. A dropout layer is used after concatenation for regularization.

#### 2.3.3. Hybrid Graph Autoencoder-Regressor Ensemble (HGARE)

A hybrid graph neural network, termed the Hybrid Graph Autoencoder-Regressor Ensemble (HGARE), was developed to predict anion conductivity in AEMs. This framework was designed to first learn chemically meaningful latent representations through unsupervised learning and subsequently refine these embeddings via supervised fine-tuning within an ensemble-learning setup to enhance stability and predictive accuracy.

**Figure 4:**
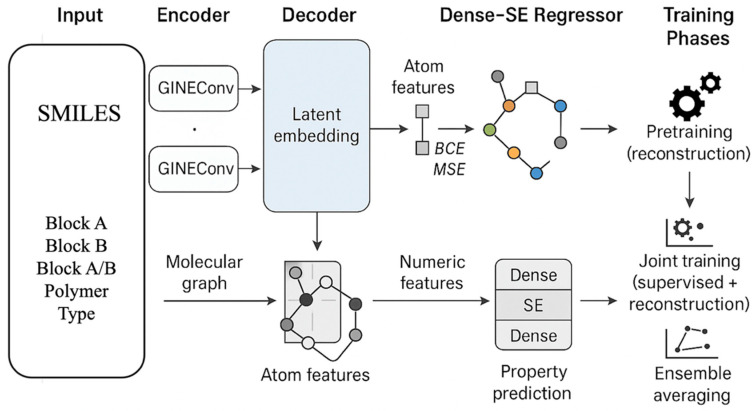
Schematic of Hybrid Graph Autoencoder-Regressor (HGARE) architecture.

The HGARE architecture comprises three principal modules as shown in Figure 4: a graph encoder, a node-feature decoder, and a Dense-SE regressor. The encoder was implemented using multiple layers of the Graph Isomorphism Network with Edge Attributes (GINEConv), each followed by batch normalization, ReLU activation, and dropout regularization. Three consecutive layers generated atom-level embeddings that were aggregated by global mean pooling to form a compact graph-level latent representation. To ensure that the encoder captured a chemically consistent latent space, a lightweight feed-forward decoder reconstructed the node features from the latent embeddings, enforcing structural awareness through a combined binary cross-entropy and mean-squared error reconstruction loss.

The Dense-SE regressor received the concatenated vector composed of the learned molecular embeddings together with hand-crafted molecular descriptors and polymer composition features, including the percentage of Block A, the percentage of Block B, and the Block A/B ratio. The regressor consisted of several dense blocks, each comprising linear, batch normalization, ReLU, and dropout layers. To enhance representation learning, a squeeze-and-excitation (SE) attention mechanism was incorporated to adaptively re-weight hidden channels, emphasizing the most informative features. The regressor output a single scalar corresponding to the predicted ionic conductivity.

Model optimization was performed in two stages. In the first stage, the encoder–decoder pair underwent denoising autoencoder pretraining using only the reconstruction losses to capture intrinsic molecular regularities. In the second stage, the pretrained encoder was coupled with the regressor and fine-tuned jointly. The joint loss function combined supervised and reconstruction components as

(4) $$L=\lambda_{sup}(\alpha L_{SmoothL1}+\left(1-\alpha \right)L_{MSE})+(1-\lambda_{sup})L_{recon}$$

where L_sup_ represents a weighted combination of Smooth L1 and mean-squared error losses, L_recon_ denotes the reconstruction loss, and *λ* controls the relative weighting between supervised and unsupervised objectives.

Each component of the HGARE architecture (AE pretraining, SE block, joint reconstruction loss, and ensemble) was later evaluated individually through ablation analysis to quantify its contribution to predictive performance. In contrast to existing hybrid graph neural network autoencoder frameworks, the proposed Hybrid Graph Autoencoder-Regressor Ensemble (HGARE) introduces several architectural and training distinctions. The key differences are as follows:

1. SE-based feature recalibration: HGARE incorporates a squeeze-and-excitation (SE) attention block that adaptively weights node embeddings—an element that is not present in standard GNN-AE hybrids.
2. Joint reconstruction–regression training: Instead of pretraining followed by isolated regression, HGARE employs dual-loss fine-tuning, enabling more stable representation learning.
3. Graph ensemble averaging: HGARE aggregates predictions across multiple random graph initializations, substantially improving robustness (as shown by ablation).
4. Tailored architecture for polymer descriptors: Unlike prior small-molecule GNN-AEs, HGARE handles block–copolymer graphs with repeating-unit expansion and cross-link representations. The HGARE framework effectively integrates the structural interpretability of graph neural networks with the representational flexibility of dense architectures, thereby leveraging both molecular topology and global physicochemical descriptors. The combination of autoencoder pretraining, joint fine-tuning, and ensemble averaging results in stable, accurate, and chemically consistent predictions of anion conductivity in AEMs. Consequently, HGARE represents a robust and generalizable modeling paradigm for polymer informatics and data-driven materials discovery.

## 3. Dataset

The dataset was further curated by reconstructing polymer structures from the original publications and generating canonical SMILES strings. We performed an additional round of manual structural curation. Each membrane was traced back to its original publication, the polymer structure was redrawn in ChemDraw Suit 23.1.2 [50], and canonical SMILES strings were generated. These smiles were then carefully checked against their backbone identity, cationic group, and block composition to ensure structural accuracy. In some cases, the original literature [35] did not provide sufficient detail to reconstruct the chemical structure. Such entries were excluded to preserve the integrity of dataset, leaving samples that could be verified. The outcome of this process is a curated dataset of 207 AEMs from 48 peer-reviewed publications found in Web of Science. This curated resource is not only internally consistent but also extends the original database by providing canonical SMILES that were not available previously. Each record includes SMILES strings of the whole polymer structure, Block A and Block B and Block A/B composition, and experimentally reported hydroxide conductivity at 80 °C. This dataset served as the input for both descriptor-based and graph-based machine learning workflows. Molecular graphs were created from SMILES using RDKit (version 2024.03.5) [51], and descriptors were computed using Mordred [6] and RDKit libraries [51]. The full dataset, including membrane names, membrane structures, and source references, is provided in Supporting Information to facilitate transparency and reproducibility (Table S1).

Molecular descriptors were computed using RDKit and the Mordred toolkit, a molecular representation method that encodes atomic composition, electric properties, topological indices, and geometric configurations of each polymer molecule, yielding an initial 1610 descriptors. All descriptors were converted to numeric format, and columns/rows with missing values were dropped to ensure clean inputs. This resulted in a dataset of 1432 descriptors per sample. Additionally, engineered features such as Block A and B compositions, Block A/B ratio, and polymer type encoded as a binary variable (0 = homopolymer, 1 = copolymer) were appended to each row to reflect microstructural context. These descriptors span a wide range of physiochemical, topological, and electrostatic properties, and they serve as the foundation for both descriptor-based supervised learning and unsupervised learning clustering described later in this work.

### External Data Curation

In addition to the primary dataset, an external set of 20 anion exchange membranes [19] was assembled to evaluate the generalization of the model under domain shift. The identification of suitable external data was nontrivial, as open-source databases for anion exchange membranes are limited and experimental data are typically scattered across individual studies with heterogeneous reporting standards. Conductivity values were curated from independent experimental reports of hydroxide conductivity measured at comparable temperatures and hydration levels. Only membranes with explicitly reported chemical structures, block compositions, and conductivity measurements were retained. All molecular structures were validated using RDKit, and entries with invalid or non-sanitizable SMILES representations were excluded. Minor inconsistencies in units and data formats were standardized, while no smoothing or imputation was applied in order to preserve the intrinsic experimental noise.

The external dataset was derived primarily from the compilation reported by Ramprasad and co-workers [19], in which several membrane chemistries were measured under multiple experimental conditions. For membranes reported more than once under comparable conditions, conductivity values were averaged to obtain a single representative value per unique chemistry, thereby avoiding overrepresentation while retaining experimental variability. The resulting dataset spans a narrow conductivity range and exhibits higher relative noise than the primary dataset, rendering correlation-based metrics such as R^2^ inappropriate for performance assessment. Consequently, model evaluation relied on absolute error-based metrics and a regressor-only domain adaptation strategy during inference. Additional details of the dataset curation and numerical distributions are provided in Supporting Information (page 2–4).

## 4. Training and Hyperparameter Optimization

For all models, we employed Optuna [52], a Bayesian optimization framework to perform hyperparameter tuning over 20 trials per model. Prior to optimization, the dataset was divided into random 90/10 train–test sets. Within the training data, a 5-fold cross-validation (CV) strategy was implemented to evaluate model generalization and mitigate overfitting during hyperparameter search.

Each Optuna trail involved training the model on the four folds and validating the remaining one. The final model configuration was retained using the best hyperparameters on the full training set and evaluated on the held-out test set.

To further assess the robustness and interoperability of the trained models, we conducted ablation analysis; in addition, SHAP (Shapley Additive exPlanations) [29] analysis was applied to evaluate feature importance and instability of trained models.

For graph neural network models including GCN and GAT, and the proposed HGARE, all implementations were developed in PyTorch 2.6 [53] and PyTorch Geometric 2.5. For the graph-based models, each polymer was reported as a molecular graph using RDKIT, where atoms were treated as nodes and bonds as edges. Node features were derived using canonical atomic features (e.g., atom number, aromaticity, and formal charge). These graphs were concatenated with the same composition features (Block A, Block B, Block A/B ratio, polymer type) to form a hybrid input for graph models. Hyperparameter tuning and model selection were performed using internal validation sets, derived from statistical random splits of training data.

We optimized models with Optuna, minimizing validation of RMSE with early stopping to avoid overfitting. During training, metrics including RMSE, mean absolute error (MAE), and coefficient of determination ($R^{2}$) were recorded using TensorBoard (version 2.20.0), and the best checkpoint from each trial was saved. The optimal hyperparameters from the best performing results were then used to retrain the model on the training set, and final results were reported on held-out test splits.

## 5. Results and Discussion

To evaluate the predictive performance of different machine learning models for estimating the ionic conductivity of AEMs, a set of descriptor-based and graph-based regressors were trained and optimized. These included XGBoost, Random Forest, and CatBoost, LightGBM, ElasticNet, and MLP regressors based on Mordred descriptors, two graph neural networks (GCN and GAT), and our HGARE model trained on molecular graphs generated from SMILES. Saliency maps combined with ablation analysis provide multi-scale interpretability, highlighting atom-level contributions and feature-level relevance [28,54]. This approach aligns with recent studies using graph-level explanations in polymer informatics [55]. The clusters discovered through t-SNE and SOM not only validate patterns of notes in prior unsupervised studies [35,36,37] but also reveal distinct structural groupings associated with conductivity that were not previously characterized.

### 5.1. Descriptor-Based Models

The Mordred featurization produced approximately 1432 molecular descriptors after data cleaning per membrane, spanning topological and electrostatic properties. However, the number of available samples was comparatively small relative to feature dimensionality, resulting in a high feature-to-sample ratio.

Such high-dimensional spaces are prone to overfitting and collinearity among descriptors, which can obscure true structure–property relationships. To address this, we employed PCA to compress the descriptor space into a set of orthogonal latent features that retain the majority of the dataset’s variance. This level of compression effectively preserved the essential physicochemical information while mitigating redundancy, noise, and potential overfitting in machine learning models.

All features were first standardized to zero mean and unit variance using z-score normalization, ensuring that descriptors with different numerical ranges contributed equally to the PCA decomposition. PCA was then applied with the explained variance threshold set to 80%, automatically selecting the smallest number of components that captured most of the data variance (Figure S2).

In our case, PCA retained eight PCs, collectively explaining 80.5% of the total variance, with PC1 alone explaining 42.4%, including a strong structural–topological gradient across the membranes, representing descriptors such as ETA, MPC, and ATS linked to molecular size, branching, and topological complexity; PC2 (12.1%) dominated by FCSP3, HybRatio, and AATS families, reflecting hybridization and polarity balance.

Subsequent components (PC3-PC8) encoded surface area, dipole correlation, and electronic dispersion effects, providing complementary information about molecular topology and charge distribution (Table 1).

This dimensionality reduction not only reduced overfitting but also improved computational efficiency during model optimization.

To further interpret the compressed feature space, Table 1 summarizes the variance explained by each PCA component along with their dominant descriptor families and physicochemical interpretation.

Using these PCA components as inputs, several descriptor-based machine learning models were trained. As summarized in Table 2, ensemble models CatBoost ($R^{2}$ = 0.8566), XGBoost ($R^{2}$ = 0.8445), and Random Forest (0.8477) practically outperformed shallow and linear baselines such ElasticNet (0.4385), confirming the nonlinearity of conductivity–structure relationships. The CatBoost model achieved the lowest prediction error (MAE = 0.0155 S/cm, RMSE = 0.0187 S/cm), indicating robust generalization after dimensionality reduction.

**Figure 5:**
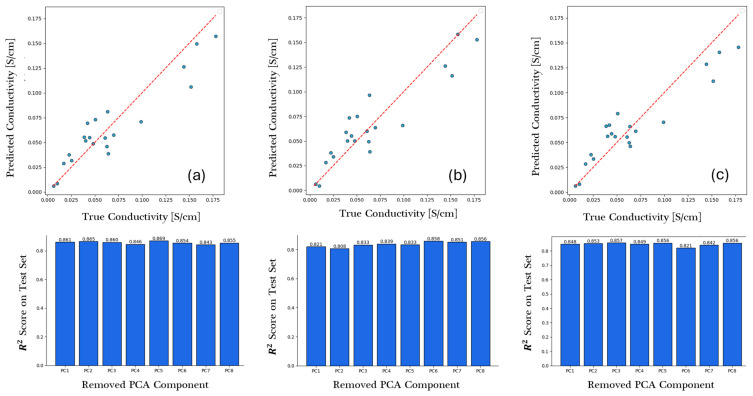
Descriptor-based models’ performance; parity plots (**top**) compare predicted and actual anion conductivity (S/cm), the dotted line represents the ideal parity line (y = x), and circles represents individual data points. And ablation analysis of plots (**bottom**) indicate how sensitive the model’s predictive accuracy is to each component space for (**a**) CatBoost, (**b**) XGBoost, (**c**) Random Forest.

The ablation analysis was performed by systemically removing each PCA component and reevaluating model performance. This method shows the relevance of each PCA component to model performance (Figure 5 Bottom figures). Among the tested algorithms, CatBoost, XGBoost, and Random Forest were selected for visualization because they consistently ranked as the best-performing descriptor-based models. Across three high-performance descriptor-based models, two complementary conductivity governing mechanism consistently emerged:

1. Macromolecular topology and polarity (PC1-PC2): These components describe overall molecular size, branching, complexity, and hydrophobic–hydrophilic balance. Their strong influence, particularly in the XGboost model, highlights the morphological continuity of hydrophilic domains and the distribution of polar functional groups primary enablers of ion transport. Well-connected polymer backbones with balanced polarity facilitate continuous ion channels, enhancing charge mobility.
2. Electronic polarization and dipolar correlation (PC4-PC6-PC7): The CatBoost and Random Forest models revealed sensitivity to these components, which capture distance-weighted dipole moments, charge delocalization, and intramolecular electrostatic coupling. These effects represent the microscopic polarization environment governing ion solvation and dynamic screening within conductive regions. Enhanced electronic flexibility and dipolar alignment promote lower activation barriers for ion hopping and diffusion.

Together, these findings reveal the structural organization and electronic polarizability and cooperatively determine anion conductivity.

### 5.2. Descriptor Selection and Correlation Analysis

Figure S1 presents a combined view of SHAP summary plots and parity plots for Catboost, XGBoost, and Random Forest.

SHAP values revealed that despite slight variation in feature rankings across models, several descriptors consistently emerged as important. Notably, ETA_shape_y, a topological descriptor related to molecular geometry, was followed closely by Block A/B ratio which qualifies the relative composition of polymer blocks. Interestingly, basic compositional parameters such as Block A and Block B also ranked highly, indicating that structural balance in the copolymer plays a crucial role in determining ionic conductivity. Other descriptors like MATS1p, ATC1p, and GATS7c reflect molecular autocorrelation and three-dimensional structural information [5], aligning with the expected structure–property relationships in ion-conducting membranes.

**Figure 6:**
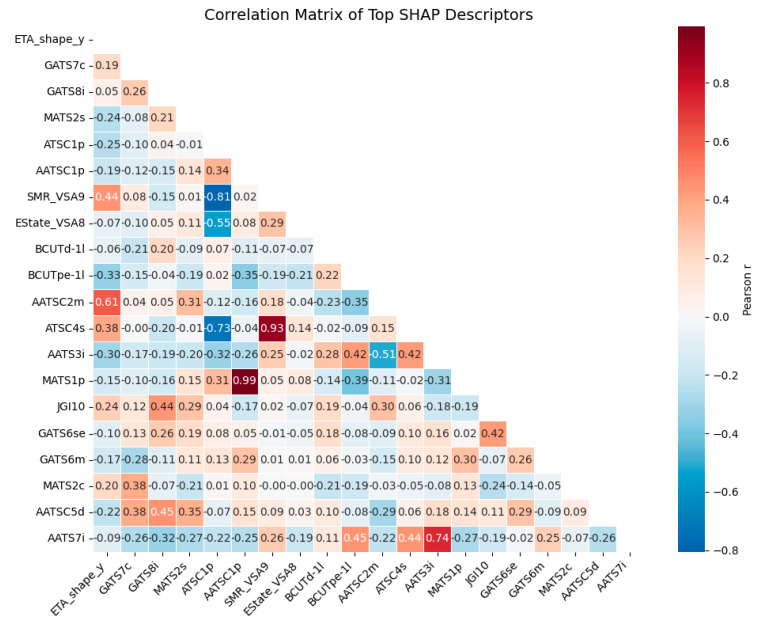
Triangle Pearson correlation matrix of top 20 SHAP-selected descriptors contributing to anion conductivity prediction in AEMs.

To further refine the descriptor space, we examined the Pearson correlation matrix of top 20 descriptors identified by SHAP across three best-performing descriptor models: CatBoost, XGBoost, and Random Forest models (Figure 6). This triangular matrix highlights pairwise correlations and reveals several strong dependencies among descriptors, indicating potential redundancy.

Notably, MATS1p and AATSC1p displayed near-perfect correlation (r ≈ 0.99), followed by SMR_VSA9 and ATSC4s (r ≈ 0.93), and AATS3i and AATS7i (r ≈ 0.74). Among these, AATSC1p with MATS1p and SMR_VSA9 with ATSC4s were deemed highly redundant, reflecting nearly identical underlying information regarding mass and electrotopological autocorrelation of atomic properties. Therefore, one descriptor from each correlated pair was excluded to reduce feature redundancy without sacrificing chemical interpretability.

On moderate correlations, the selection retained the one that was both higher in SHAP importance and chemically more interpretable. For example, MATS1p was retained over AATSC1p, as it provided stronger SHAP contributions (Figure S2) while still representing molecular autocorrelation.

Other moderate correlations, such as between ETA_shape_y and AATSC2m (r ≈ 0.61), suggest that shape anisotropy and molecular autocorrelation are partially linked but still capture distinct physical phenomena; specifically, ETA_shape_y describes molecular three-dimensional compactness, while AATSC2m encodes the averaged mass distribution weighted by atom-pair distances. Hence, both remain as they represent complementary structural and topological factors influencing ion transport.

Through this two-step process, SHAP-based ranking followed by correlation-based pruning, we reduced the feature set from the original 20 to a more compact and informative set of 17 descriptors. These retained descriptors balance predictive performance with interoperability, ensuring that the final model captures diverse, non-redundant structural and structural features relevant to anion conductivity.

To enhance the clustering analysis and more directly capture the relationship between structure and performance, we incorporated the target variable (membrane anion conductivity (S/cm)) into the features set used for clustering. Additionally, a derived numeric feature test was introduced. This variable was derived using the following formula:

(5) $$\mathrm{test}=\left\{\begin{array}{cc} 1, & if\;conductivity\geq 0.05\;S/\mathrm{cm}\\ -1, & otherwise \end{array}\right.$$

This formulation provides a coarse but meaningful distinction between high and low conductivity membranes in our dataset. Including this binary label in the clustering pipeline allows us to assess whether structure-based clusters correspond to performance categories. The distribution of anion conductivity values is shown Figure S3. This plot reveals a right-skewed distribution, with a natural cutoff near 0.05 S/cm, supporting our use of this threshold in defining the classification label. After identifying a refined set of informative, minimally correlated descriptors, we sought to explore whether these features could capture latent structural patterns related to ionic conductivity. To this end, we applied unsupervised learning techniques to visualize the chemical space [56] of AEMs and investigate whether distinct performance-relevant subgroups could be identified.

### 5.3. Clustering of Descriptor Space

**Figure 7:**
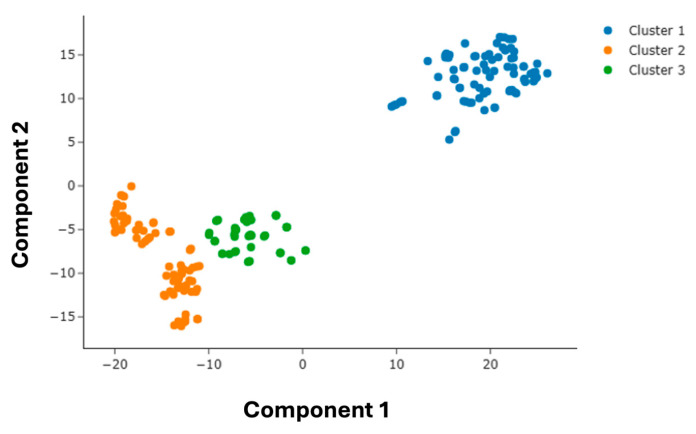
Clustering of AEM samples using KMeans in two-dimensional descriptor space.

To explore the structure–performance relationship from an unsupervised perspective, KMeans clustered in a reduced two-dimensional feature space obtained via t-SNE using the selected molecular descriptors. As shown in Figure 7, the clustering separated the AEMs into three distinct groups. Cluster 1, positioned at the top, contained the majority of high-conductivity membranes, forming tight and isolated groups. In contrast, clusters 2 and 3, situated to the bottom, represented two subtypes of low-conductivity membranes, suggesting that multiple structural motifs can independently contribute to poor performance.

To quantitatively assess the grouping structure suggested by clustering, we computed silhouette coefficients and Davies–Bouldin indices in PCA-reduced descriptor space. Both metrics showed local optima at K = 3, with a positive silhouette score (0.32) and a minimum in the Davies–Bouldin index. These results justify the selection of three clusters by indicating a balanced combination of intracluster compactness and intercluster separation.

**Figure 8:**
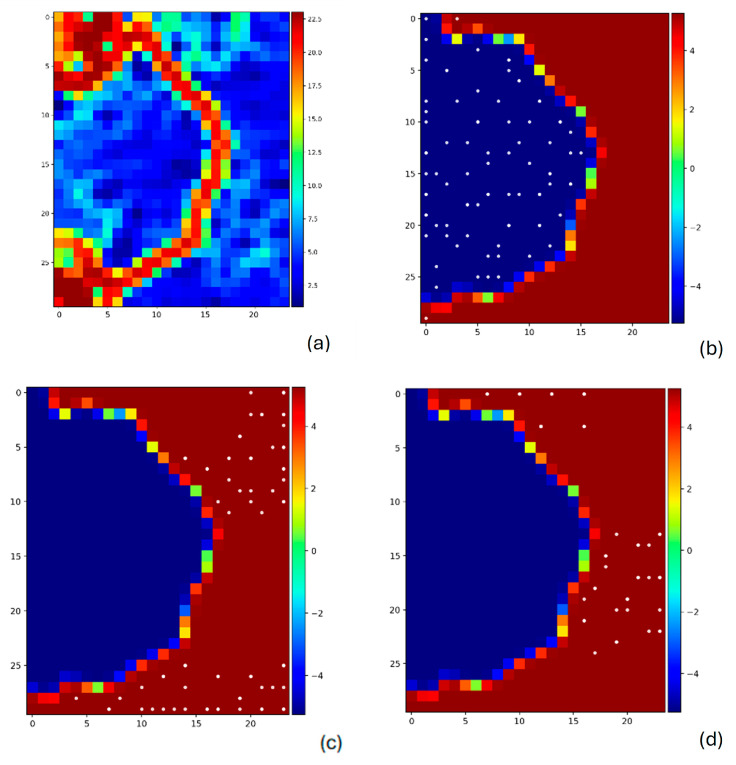
SOM figures, (**a**) SOM U-Matrix showing topological distances between mapped units; darker regions indicate higher dissimilarly. (**b**) Cluster 1, (**c**) cluster 2, and (**d**) cluster 3 are shown in white circles.

We employed a Self-Organizing Map (SOM) using JMP-Fastman (version 0.6) [57] to visualize the component contribution of the selected descriptors. In this analysis, we included the binary test feature (test = 1 if conductivity > 0.05 S/cm; −1 otherwise), which served as a classification component to highlight differences between high- and low-conductivity membranes. SOMs were trained (10 × 10,500 iterations, decaying learning rate from 0.5 to 0.01). The SOM projected high-dimensional descriptor data onto a two-dimensional map while preserving topological relationships. The resulting SOM U-matrix revealed a clear segmentation of feature space, with well-defined distance boundaries indicating distinct regions of similarity among the data samples. In this U-matrix, darker areas indicate higher dissimilarity between nodes and stronger cluster boundaries, while lighter areas denote closely related samples. When coloring the test variable, the SOM demonstrated a strong separation between high- and low-conductivity membranes, with high-performance samples forming a compact and isolated region on the map. Figure 8b represents cluster 1, which corresponds to the high conductivity membranes to exhibit consistent molecular patterns and physicochemical attributes.

To further interpret the SOM clusters, we examined component (feature) contribution analysis across the map, Block A/B ratio was substantially higher in the region associated with high conductivity (cluster 1), confirming that membranes with a greater fraction of hydrophilic block are structurally optimized for ion transport. In many AEMs, Block A often corresponds to hydrophilic moieties such as quaternary imidazolium or ammonium functional groups. These positively charged groups not only attract water but also enable efficient transport of hydroxide ions (${OH}^{-}$) by supporting the formation of ion conduction channels.

TopoPSA highlights the importance of polar functional group exposure in facilitating ion conduction, which was elevated in the high-conductivity cluster. PEOPE_VSA11 also exhibited a notable contribution in cluster 1 compared to cluster 2. This descriptor represents the partial equalization of orbital electronegatively (PEOE)-based van der Waals surface area within a specific charge range, effectively quantifying regions of moderate positive electrostatic potential on the molecular space.

**Figure 9:**
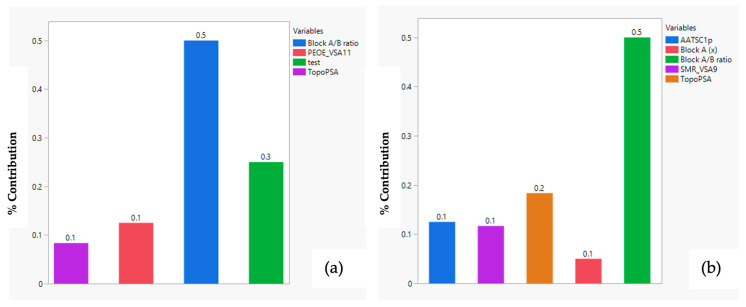
Bar plots comparing (**a**) cluster 1 to 2, (**b**) cluster 2 to 3 average descriptor values.

Bar plots summarizing descriptor values with each SOM-identified cluster reinforced these (Figure 9).

Across PCA, SHAP, and clustering analyses, a common trend emerged; molecular topology, hydrophilic hydrophobic balance, and electronic polarization consistently differentiate high- from low-conductivity AEMs.

Based on the insights gained from PCA, SHAP, saliency analysis, and SOM, a set of design rules emerges that can guide the selection and engineering of high-conductivity structures.

1. Extended molecular shape (e.g., high values of the descriptor ETA_shape_y), suggesting that elongated or extended polymer architectures may enhance ion transport by facilitating continuous ionic domains.
2. Elevated polar surface area and charge-rich regions (e.g., descriptors such as topoPSA, PEOE_VSA11, ESTATE_VSA8) indicating that polar/electronic surface features favor formation of hydrophilic, ion-conducting pathways.
3. Intermediate values of two-dimensional autocorrelation/topological descriptors (e.g., GATS- and MATS-type descriptors reflecting a balance between rigidity/flexibility and charge delocalization, which may optimize microstructure and ionic mobility).

These rules are intended to be heuristic guidance (not hard thresholds) for selecting candidates with high likelihood of good conductivity in future polymer design.

### 5.4. Graph-Based Models

Graph neural networks were trained directly to learn from topological structures of molecules. The GCN model achieved the highest performance across all models, with a test R^2^ of 0.8807 and MAE of 0.0143, demonstrating good performance (Table 2). The GAT model yielded lower R^2^ of 0. 8186, suggesting that attention-based aggregation did not offer a performance advantage in this context. The GAT model’s performance may stem from overfitting due to the increased parameter complexity from the fact that the learned attention weights provided limited added value over simple aggregation in this dataset.

**Figure 10:**
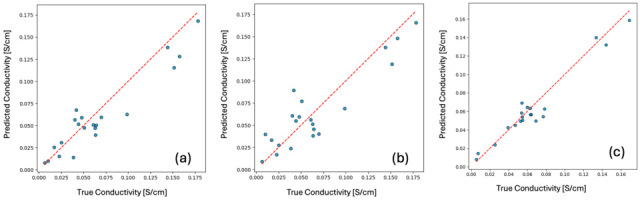
Parity plot for test set in (**a**) GCN model, (**b**) GAT model; (**c**) HGARE achieves near-perfect parity, supporting its high predictive accuracy. The dotted line represents the ideal parity line (y = x), and circles represents individual data points.

The parity plot for GCN (Figure 10a) shows close alignment between predicted and true conductivity values for both test and train sets, validating its robustness. The strong predictive capacity of GCN highlights the effectiveness of graph-based learning in capturing complex structure–property relationships. For GCN, the optimal parameters were input dimension = 74, hidden dimension = 256, learning rate = $8.42\times 10^{-4}$, batch size = 6, and 500 training epochs. For the GAT model, the best configuration employed input dimension = 74, hidden dimension = 128, learning rate = $1.19\times 10^{-3}$, batch_size = 8, and 1500 training epochs.

Building on these baselines, the proposed HGARE achieved a substantial improvement by integrating graph autoencoder pretraining, joint supervised fine-tuning, and ensemble averaging. This model achieved a test R^2^ of 0.9460 and MAE of 0.0070, outperforming all models. The parity plot in Figure 10c shows an almost perfect diagonal alignment between predicted and true values, confirming the model’s accuracy and generalization capability. The best hyperparameter settings obtained via random search are hidden dimension = 224, encoder layers = 4, encoder dropout = 0.08, autoencoder learning rate = $1\times 10^{-3}$, AE weight decay = $1\times 10^{-5}$, AE epochs = 120, batch size = 48, main learning rate = $7\times 10^{-4}$, weight decay = $5\times 10^{-4}$, dropout = 0.20, head-hidden = 256, α = 0.60, supervised loss weight = 0.92, reconstruction weight = 0.08, cosine annealing scheduler ($T_{O}$ = 120, Tmult = 2), and SWA = 0.33.

These results confirm that HGARE architecture outperformed both descriptor-based models (CatBoost, XGBoost) and conventional GNNs by capturing complex structure–property dependencies through its hybrid unsupervised–supervised training and ensemble strategy. This establishes HGARE as a state-of-the-art framework for molecular anion conductivity prediction.

### 5.5. Ablation Study of HGARE Model Component

To assess the contribution of each component within HGARE, we conducted a systematic ablation study following the reviewer’s recommendation. Four model variants were evaluated by independently removing (i) the autoencoder (AE) pretraining, (ii) the squeeze-and-excitation (SE) attention block, (iii) the reconstruction loss used during joint fine-tuning, and (iv) the ensemble averaging. A GCN + MLP baseline was also included for comparison. All variants were trained under identical conditions and using the same data split as the full HGARE model to ensure fair comparison.

Table 3 summarizes the results. The full HGARE achieves the highest accuracy (R^2^ = 0.948), demonstrating the effectiveness for predicting ionic conductivity. Removing AE pretraining produces almost no degradation, which is expected given the small dataset size. Eliminating the SE block reduces performance to 0.940, confirming that the feature recalibration contributes meaningfully. Removing the reconstruction loss causes a mild decrease in accuracy (R^2^ = 0.940), confirming that the feature recalibration contributes meaningfully. Removing the reconstruction loss causes a mild decrease in accuracy (0.947), suggesting that reconstruction offers weak regularization. In contrast, removing the ensemble produces a substantial drop (R^2^ = 0.907), indicating that ensembling is critical for variance reduction and stability. The GCN + MLP baseline (R^2^ = 0.942) performs well but remains below the full HGARE.

Overall, the ablation results validate the necessity of SE-based feature weighting and ensemble averaging within HGARE, while clarifying that AE pretraining offers minimal benefit for the dataset. These findings directly address the reviewer’s concern regarding architectural complexity and demonstrate which components meaningfully improve model performance.

### 5.6. Saliency Maps

**Figure 11:**
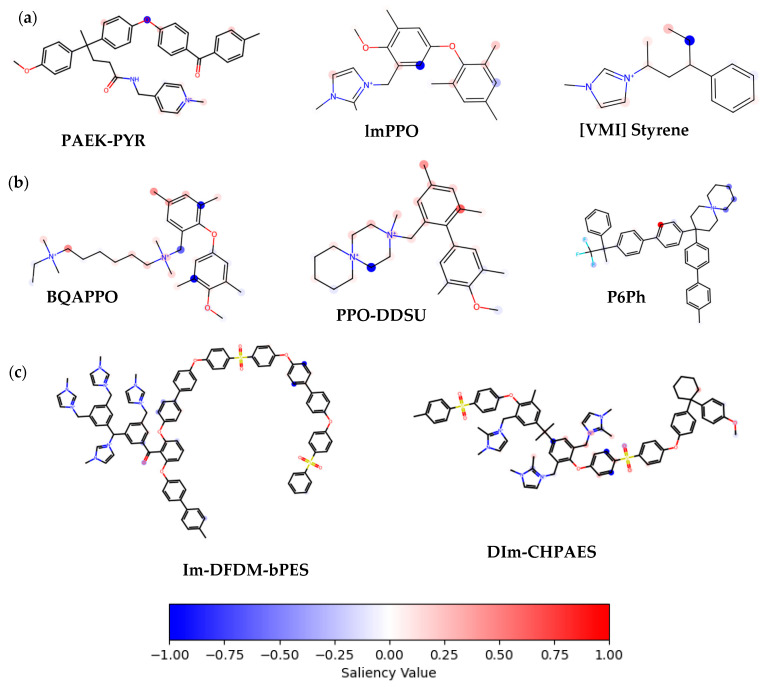
Saliency maps of representative anion exchange membranes (AEMs), grouped by predicted anion conductivity at 80 °C. Red and blue colors indicate positive and negative contributions, respectively, to the predicted conductivity, normalized across each molecule. Molecules are grouped into three conductivity classes: (**a**) low (<0.06 S/cm), (**b**) moderate (0.06–0.15 S/cm), and (**c**) high (>0.20 S/cm).

To investigate the interpretability of our model and understand how structural features influence anion conductivity predictions, we generated atom-level saliency maps using gradient-based attribution, a widely adopted technique for GNN explainability [54], for selected representative molecules. Figure 11 presents a qualitative comparison of these molecules, grouped by their predicted anion conductivity at 80 °C. The saliency values were obtained through gradient-based attribution and are visualized as heatmaps overlaid on each molecular structure. Red colors represent atoms with a positive contribution to the predicted conductivity, while blue tones indicate negative contributions. All saliency values are normalized per molecule. Similar methods have been explored in recent studies of explainable GNNs, particularly those using gradient-based attribution techniques [54].

Across low-conductivity parts (Figure 11a), nitrogen atoms, particularly those forming quaternary ammonium groups, are consistently assigned to negative saliency values (blue), whereas surrounding oxygen atoms (e.g., in ether, ester, or carbonyl functionalities) appear red, indicating a positive contribution. This pattern implies that while cationic groups are required for conductivity, their local structural context, such as spatial, isolation, limited hydration, or steric hindrance, may reduce their effectiveness. For example, the imidazolium group in [VMI] Styrene shows localized blue shading, potentially reflecting insufficient hydrophilicity or poor connectivity to conduction pathways.

In contrast, high-conductivity AEMs (Figure 11), such as lm-DFDM-bPES and Dlm-CHPAES, display pronounced red saliency values near sulfonyl oxygen atoms, ether linkers, and flexible aliphatic segments. Interestingly, the positively charged imidazolium groups in these structures show neutral to slightly blue saliency, implying that their conductive benefits arise more from favorable interaction with surrounding hydrophilic domains than from charged centers alone. The extended conjugation and high segmental mobility in these molecules likely continue to enable efficient ion transport, consistent with known design principles in high-performance AEMs.

These observations align with prior experimental insights, where conductivity is enhanced not solely by the presence of cationic groups but also by their spatial distribution, hydration environment, and their polymer chain flexibility. Our model appears to capture these nuanced relationships, confirming that their predictions are grounded in chemically interpretable structure–property associations.

## 6. Conclusions

We present a hybrid machine learning framework that integrates unsupervised clustering, descriptor-based modeling, and graph neural networks to predict and interpret ionic conductivity in AEMs.

Among all tested models, the Hybrid Graph Autoencoder-Regressor Ensemble (HGARE) achieved the highest accuracy and robustness. By jointly optimizing a denoising graph autoencoder with a dense squeeze–excitation regressor and applying ensemble averaging across multiple seeds, HGARE effectively captures both local atomic interaction and global compositional features.

For descriptor based-models, PCA was applied to reduce dimensionality and mitigate multicollinearity among 1400+ Mordred descriptors while retaining 80% of the variance. Ablation analysis was then performed on these components to examine how each descriptor group influences ionic conductivity. The result revealed that structural and topological and electrostatic components were primary contributors.

To explore structure–performance relationships beyond prediction, we applied unsupervised learning using t-SNE, KMeans, and Self-Organizing Maps (SOMs). This analysis revealed three distinct membrane clusters, including two low-conductivity subtypes and one high-performance group characterized by flexible, polarizable, and hydrophilic-rich architectures. The clustering results are consistent with ablation analysis and saliency mapping, highlighting how segmental flexibility, local charge distribution, and hydrophilic content collectively enable efficient ion transport. Saliency highlights the gradients of model prediction with respect to node features, revealing chemically meaningful motifs such as quaternary ammonium groups, imidazolium cations, and aromatic backbones that form ion-conducting domains.

This framework not only enables accurate prediction but also chemically interpretable insights, bridging the gap between data-driven predictions and rational AEM design. This combined approach offers a powerful tool for rational AEM design and can be extended to other polymer property predictions. In the future, the framework can be extended to predict multiple properties simultaneously using multitask learning [57], accelerating the discovery of high-performance AEMs. Relevant targets include chemical stability under alkaline conditions, water uptake, swelling ratio, ion exchange capacity (IEC), hydrophilicity distribution, and mechanical durability, each of which contributes to real-world AEM performance. Achieving this requires a curated dataset where these properties are inconsistently measured across identical or structurally comparable membranes. With such data, the proposed HGARE architecture can be adapted to a multitask setting by sharing the graph-based encoder while introducing separate prediction heads for each property. Experimental synthesis and in-plane conductivity measurements of these candidates are important directions for future collaboration with synthetic groups.

## Figures and Tables

**Table 1:** Variance contribution of the top eight principal components obtained from the Mordred descriptor matrix.

Principal Component	Explained Variance (%)	Dominant Descriptor Families	Physicochemical Interpretation
**PC1**	42.41	ETA, MPC, ATS, BertzCT, Xp	Overall molecular size branching and topological complexity, dominant structural factor governing ion transport.
**PC2**	12.13	FCSP3, hybRatio, AATS, GATS, AETA	Degree of hybridization, polarity, and carbon saturation influencing charge delocalization and hydrophobic–hydrophilic balance.
**PC3**	6.77	ATSC, VSA_Estate, AATS	Electronic surface area and atomic electronegativity effects on local charge distribution.
**PC4**	5.71	GATS, AATSC, MATS	Short-range autocorrelation of atomic properties, dipole-included and intramolecular interaction patterns.
**PC5**	4.19	AATSC, MATS, GATS	Weighted descriptors of surface polarity and dispersion interactions.
**PC6**	3.67	GATS, MATS, AATSC	Distance-weighted dipole descriptors linked to electronic polarizations.
**PC7**	3.36	GATS, AATSC, MATS	Medium-range spatial autocorrelation descriptors, topology-dependent polarizability.
**PC8**	2.30	AATSC, MATS, AMID, JGI	Connectivity indices and electronic delocalization parameters associated with charge transport continuity.

**Table 2:** Performance summary of all models.

Model	Type	R^2^ (Test)	RMSE (Test)	MAE (Test)	Notes
HGARE	Graph-based	0.9460	0.0093	0.0070	Best overall model
GCN	Graph-based	0.8807	0.0178	0.0143	Good performance
GAT	Graph-based	0.8186	0.0175	0.0210	Weaker than GCN
XGBoost	Descriptor-based	0.8445	0.0195	0.0158	Good Performance
Random Forest	Descriptor-based	0.8477	0.0193	0.0161	Good performance
CatBoost	Descriptor-based	0.8566	0.0187	0.0155	Best descriptor model
ElasticNet	Descriptor-based	0.4285	0.0373	0.0295	Weak performance
LightGBM	Descriptor-based	0.7815	0.0231	0.0186	Weaker performance
MLP	Descriptor-based	0.8340	0.0201	0.0167	Good performance

**Table 3:** Ablation study results for HGARE.

Variant	R^2^	RMSE	MAE
Full HGARE	0.9475	0.00919	0.00653
No AE	0.9483	0.000912	0.00687
No SE	0.9403	0.00980	0.00758
No Recon	0.9467	0.00926	0.00691
No Ensemble	0.9072	0.01222	0.00976
GCN + MLP Baseline	0.9419	0.000966	0.00732

## Data Availability

All datasets used in this study, including SMILES string used for producing Mordred descriptors and graph models, are in modified_data_final.csv. The descriptors used for descriptor models are available in full_descriptor_data.csv. Both files are included in the data/folder of the repository. All scripts for models are in the src/folder and notebook/folder. The corresponding source code and data for this work are available online at https://github.com/Pegahnn/Conductivity_AEM/ (accessed on 19 October 2025).

## Supplementary Materials

The following supporting information can be downloaded at: https://www.mdpi.com/article/10.3390/membranes16010012/s1. Figure S1: SHAP summary plots (right) highlighting top molecular descriptors contributing to conductivity predictions for (a) CatBoost, (b) XGBoost, (c) Random Forest. Figure S2: Explained variance curve for PCA transformation of Mordred descriptors. The first eight components capture approximately 80% of the total variance. Figure S3: Kernel density estimate (KDE) plot showing the distribution of membrane anion conductivity values (target variable). The peak near 0.05 S/cm supports the threshold used to define the binary test variable (test = 1 if conductivity < 0.05; −1 otherwise) for performance-aware clustering. Figure S4: Absolute prediction error as a function of experimental anion conductivity for the external validation dataset following regressor-only domain adaptation. Each point corresponds to a single polymer system. The absence of systematic error growth across the conductivity range indicates stable numerical behavior under domain shift. Table S1: This dataset is used in both Graph models and descriptor-based models SMILES representation of these polymer structures used to produce molecular descriptors [58,59,60,61,62,63,64,65,66,67,68,69,70,71,72,73,74,75,76,77,78,79,80,81,82,83,84,85,86,87,88,89,90,91,92,93,94,95,96,97,98,99,100,101,102,103,104,105].
