# Supplementary material for: Uncovering Structure–Conductivity Relationships in Anion Exchange Membranes (AEMs) Using Interpretable Machine Learning
Source: Membranes (Basel). 2025 Dec 31;16(1):12. doi: 10.3390/membranes16010012 (PMC12843994; doi:10.3390/membranes16010012)
Supplement: Supplementary file 1 [file membranes-16-00012-s001.zip › membranes-3988847-supplementary.pdf]

Supporting Information for

# Uncovering Structure–Conductivity Relationships in AEMs Using Interpretable Machine Learning

P. Naghshnejad [a], D. Das [b], J. A. Romagnoli [a *], R. Kumar [b], J. Chen, [c]

[a] Department of Chemical Engineering, Louisiana State University, Baton Rouge, LA 70803

[b] Department of Chemistry, Louisiana State University, Baton Rouge, LA 70803

c Department of Computer Science, Louisiana State University, Baton Rouge, LA 70803

* Contact information : Professor Jose A. Romagnoli, jose@lsu.edu

This supporting information contains complete curated dataset used for both descriptor-based and graph-based models. Each entry includes canonical SMILES string (used to generate descriptors and molecular graphs of each membrane, Block compositions, and experimentally reported hydroxide conductivity at 80°C. It also includes SHAP summary plots highlighting the most influential molecular descriptors contribution to anion conductivity predictions across different models. It also includes principal component analysis (PCA) plots for dimensionality reduction. Additionally, Kernel Density Estimate (KDE) visualizations are included.

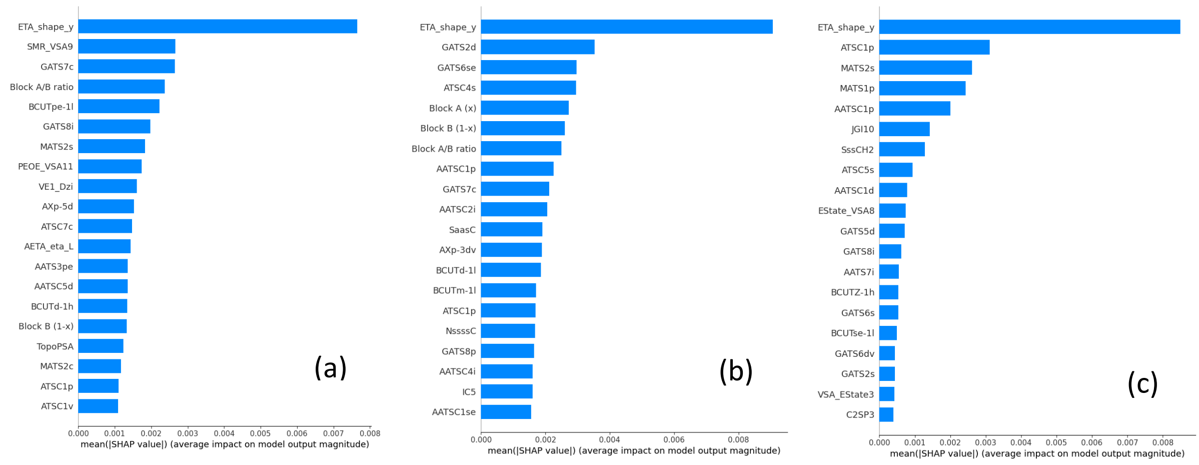


**Figure S1.** SHAP summary plots(right) highlighting top molecular descriptors contributing to conductivity predictions for (a) CatBoost, (b) XGBoost, (c) Random Forest.

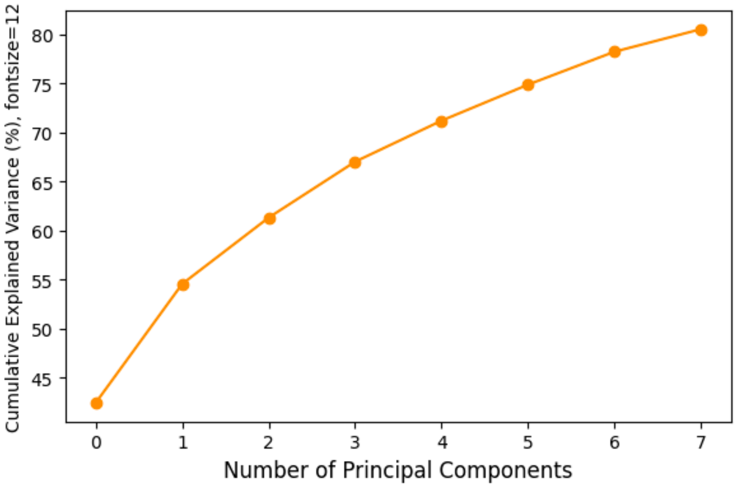


**Figure S2.** Explained variance curve for PCA transformation of Mordred descriptors. The first eight components capture approximately 80% of the total variance

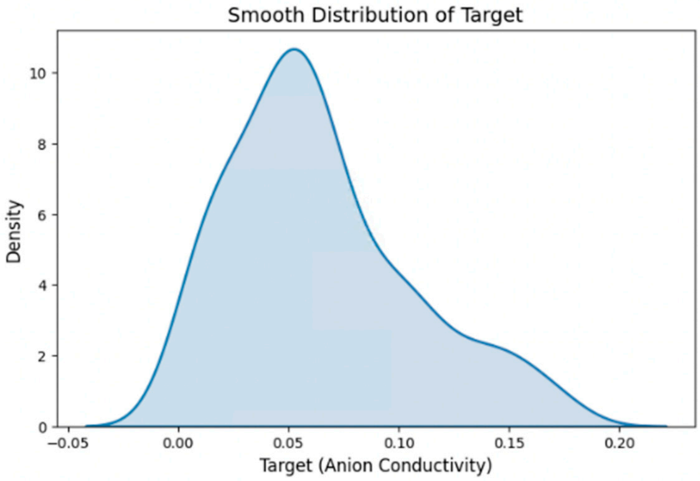


**Figure S31.** Kernel density estimate (KDE) plot showing the distribution of membrane anion conductivity values (target variable). The peak near 0.05 S/cm supports the threshold used to define the binary test variable (test=1 if conductivity $< 0.05$; -1 otherwise) for performance-aware clustering.

## External Validation and Domain Adaptation Analysis

An external dataset comprising 20 polymer systems was employed to evaluate the robustness of the trained graph-based model under domain shift. The dataset spans a narrow range of experimentally measured anion conductivity values, with limited variance and a small sample size relative to the training data. Such characteristics impose inherent statistical constraints on model evaluation, particularly with respect to correlation-based goodness-of-fit metrics. In this regime, the coefficient of determination ($R^2$) is known to be highly sensitive to minor systematic offsets and experimental noise, often yielding misleading or negative values

even when prediction errors are small in absolute terms. For this reason, absolute error–based metrics were adopted as the primary indicators of predictive performance for the external validation.

To mitigate overfitting while allowing for limited domain adaptation, a regressor-only fine-tuning strategy was applied during inference. In this approach, the graph encoder responsible for extracting molecular-level representations was kept fixed, thereby preserving the learned chemical features from the original training set. Only the final regression layers were fine-tuned using the external dataset, enabling the model to adjust for differences in scale and offset between datasets without altering the underlying structural representations. Given the small size of the external dataset, no train–test splitting was performed; instead, performance was assessed on the full dataset following adaptation, consistent with common practice for low-sample external validation.

Using this strategy, the model achieved a mean absolute error of 0.019 S $cm^{-1}$ and a root mean squared error of 0.025 S $cm^{-1}$ on the external dataset. These error magnitudes are comparable to the intrinsic variability of the experimental measurements, indicating that the predictions remain numerically stable despite the presence of domain shift. Inspection of the absolute prediction errors as a function of experimental conductivity reveals no systematic amplification of error across the target range, suggesting that the model does not exhibit strong heteroscedastic behavior under these conditions. While rank-based correlation metrics were explored during preliminary analysis, their instability across repeated evaluations confirmed that rank ordering could not be reliably inferred from the available data. Consequently, absolute error–based metrics were retained as the most appropriate and interpretable measures of model performance for this external validation.

Overall, the external validation results demonstrate limited but meaningful generalization, with prediction errors constrained to experimentally relevant magnitudes. The observed performance is consistent with the statistical ceiling imposed by the small size and narrow dynamic range of the external dataset and highlights the importance of cautious metric selection when evaluating machine learning models under data-limited conditions.

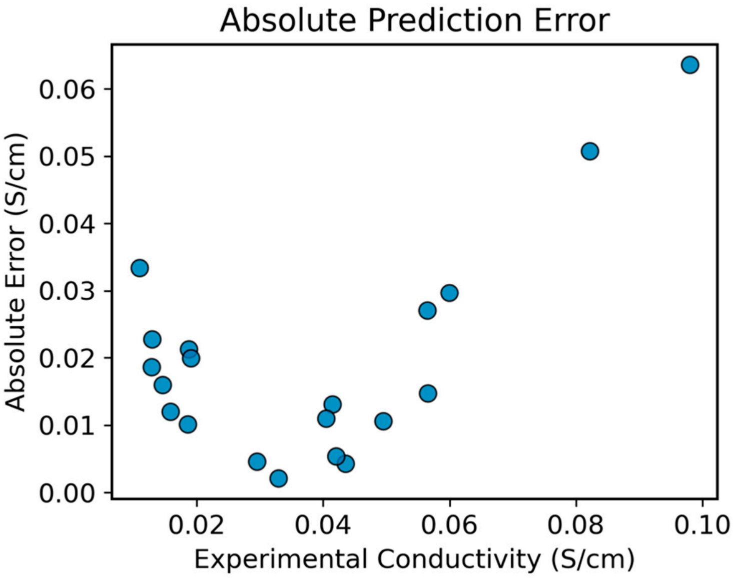


Figure S 4. Absolute prediction error as a function of experimental anion conductivity for the external validation dataset following regressor-only domain adaptation. Each point corresponds to a single polymer system. The absence of systematic error growth across the conductivity range indicates stable numerical behavior under domain shift.

**Table S2.** This dataset is used in both Graph models and descriptor-based models SMILES representation of these polymer structures used to produce molecular descriptors**.**

| Membrane Name | Block A (x) | Block B (1-x) | Block A/B ratio | Polymer type | Membrane Anion Conductivity [S/cm] | IEC (mmol $g^{-1}$ ) | Measurement Geometry | Hydration / RH conduction |
|---|---|---|---|---|---|---|---|---|
| PAEK-PYR50[1] | 0.198 | 0.802 | 0.247 | 1 | 0.00318 | 0.34-1.29 | In-plane four-probe AC impedance; BEKKTEC H cel; electrode spacing | Membranes exchanged to OH- and immersed in DI water>30 h |
| PAEK-PYR75[1] | 0.502 | 0.498 | 1.008 | 1 | 0.00616 | | | |
| PAEK-PYR100[1] | 0.7581 | 0.2419 | 3.134 | 1 | 0.01026 | | | |
| PAEK-PYR125[1] | 0.967 | 0.033 | 29.303 | 1 | 0.01156 | | | |
| Im-PEEK-15[2] | 0.15 | 0.85 | 0.176 | 1 | 0.017 | 0.90-1.54 | Not reported | Not reported |
| Im-PEEK-10[2] | 0.1 | 0.9 | 0.111 | 1 | 0.028 | | | |
| Im-PEEK-05[2] | 0.05 | 0.95 | 0.053 | 1 | 0.026 | | | |
| BPPO-MDPA-4[3] | 0.428 | 0.572 | 0.748 | 1 | 0.06618 | 0.48-1.62 | In-plane four-probe; Autolab | Fully hydrated |
| BPPO-MDPA-3[3] | 0.3135 | 0.6865 | 0.4567 | 1 | 0.06076 | | | |
| BPPO-MDPA-2[3] | 0.2266 | 0.7734 | 0.293 | 1 | 0.05385 | | | |

| | | | | | | | | |
|---|---|---|---|---|---|---|---|---|
| BPPO-MDPA-1[3] | 0.103 | 0.897 | 0.1148 | 1 | 0.04368 | | PGSTAT 30; cell dipped in water | |
| PES-3-QA[4] | 0.758 | 0.242 | 3.127 | 1 | 0.04859 | 1.1-1.9 | In-plane-two-point probe; 1 cm electrode spacing; cross-sectional area from membrane width and thickness | Fully-hydrated-membranes immersed in DI water |
| PES-4-QA[4] | 0.759 | 0.241 | 3.158 | 1 | 0.05093 | | | |
| PES-6-QA[4] | 0.757 | 0.243 | 3.113 | 1 | 0.06283 | | | |
| PES-8-QA[4] | 0.756 | 0.244 | 3.103 | 1 | 0.05171 | | | |
| PES-12-QA[4] | 0.753 | 0.247 | 3.049 | 1 | 0.04156 | | | |
| Im-DFDM-bPES-x7y32[5] | 0.821 | 0.179 | 4.587 | 1 | 0.0988 | Not reported | Not reported | Not reported |
| Im-DFDM-bPES-x7y24[5] | 0.774 | 0.226 | 3.425 | 1 | 0.07153 | Not reported | Not reported | Not reported |
| Im-DFDB-bPES-x4y20[5] | 0.833 | 0.167 | 4.988 | 1 | 0.06312 | Not reported | Not reported | Not reported |
| Im-DFDB-bPES-x4y15[5] | 0.789 | 0.211 | 3.739 | 1 | 0.04667 | Not reported | Not reported | Not reported |
| DIm-CHPAES[6] | 0.5 | 0.5 | 1 | 1 | 0.07 | Not reported | Not reported | Not reported |
| Im-CHPAES[6] | 0.5 | 0.5 | 1 | 1 | 0.0419 | Not reported | Not reported | Not reported |
| DIm-PAES[6] | 0.5 | 0.5 | 1 | 1 | 0.0611 | Not reported | Not reported | Not reported |
| Im-PAES[6] | 0.5 | 0.5 | 1 | 1 | 0.0767 | Not reported | Not reported | Not reported |

| | | | | | | | | |
|---|---|---|---|---|---|---|---|---|
| DIm-PSF[6] | 0.5 | 0.5 | 1 | 1 | 0.0526 | Not reported | Not reported | Not reported |
| Im-PSF[6] | 0.5 | 0.5 | 1 | 1 | 0.0654 | Not reported | Not reported | Not reported |
| PAES-5-IMPPO[7] | 0.05 | 0.95 | 0.053 | 1 | 0.0337 | Not reported | Not reported | Not reported |
| PAES-10-IMPPO[7] | 0.1 | 0.9 | 0.111 | 1 | 0.0549 | Not reported | Not reported | Not reported |
| PAES-12.5-IMPPO[7] | 0.125 | 0.875 | 0.143 | 1 | 0.0635 | Not reported | Not reported | Not reported |
| PAES-15-IMPPO[7] | 0.15 | 0.85 | 0.176 | 1 | 0.0788 | Not reported | Not reported | Not reported |
| TrimPES-0.2[8] | 0.167 | 0.833 | 0.2 | 1 | 0.0811 | Not reported | Not reported | Not reported |
| TrimPES-0.4[8] | 0.286 | 0.714 | 0.4 | 1 | 0.12 | Not reported | Not reported | Not reported |
| ImPES-0.4[8] | 0.286 | 0.714 | 0.4 | 1 | 0.0576 | Not reported | Not reported | Not reported |
| Bz-PBI-OBBA-OH[9] | 0.5 | 0.5 | 1 | 0 | 0.00684 | Not reported | Not reported | Not reported |
| Bz-PBI-IPA-OH[9] | 0.5 | 0.5 | 1 | 0 | 0.00186 | Not reported | Not reported | Not reported |
| MM-PES-1-1.5[10] | 0.4 | 0.6 | 0.67 | 1 | 0.054 | Not rep | Not reported | Not reported |

| | | | | | | | | |
|---|---|---|---|---|---|---|---|---|
| | | | | | | ort ed | | |
| MM-PES-1.5-1[10] | 0.6 | 0.4 | 1.5 | 1 | 0.0783 | Not reported | Not reported | Not reported |
| QPVK-12h[11] | 0.078 | 0.922 | 0.085 | 0 | 0.00491 | Not reported | Not reported | Not reported |
| QPVK-24h[11] | 0.159 | 0.841 | 0.189 | 0 | 0.00639 | Not reported | Not reported | Not reported |
| QPVK-36h[11] | 0.251 | 0.749 | 0.335 | 0 | 0.00804 | Not reported | Not reported | Not reported |
| QPVK-48h[11] | 0.318 | 0.682 | 0.466 | 0 | 0.01 | Not reported | Not reported | Not reported |
| QPVK-60h[11] | 0.353 | 0.647 | 0.546 | 0 | 0.0103 | Not reported | Not reported | Not reported |
| [VBI] Styrene A[12] | 0.357 | 0.643 | 0.555 | 1 | 0.0493 | Not reported | Not reported | Not reported |
| [VBI] Styrene B[12] | 0.286 | 0.714 | 0.4 | 1 | 0.0294 | Not reported | Not reported | Not reported |
| [VBI] Styrene C[12] | 0.222 | 0.778 | 0.285 | 1 | 0.0261 | Not reported | Not reported | Not reported |
| [VMI] Styrene D[12] | 0.455 | 0.545 | 0.835 | 1 | 0.0279 | Not reported | Not reported | Not reported |
| [VMI] Styrene E[12] | 0.417 | 0.583 | 0.715 | 1 | 0.0206 | Not reported | Not reported | Not reported |

| | | | | | | | | |
|---|---|---|---|---|---|---|---|---|
| [VMI] Styrene F[12] | 0.333 | 0.667 | 0.499 | 1 | 0.0146 | Not reported | Not reported | Not reported |
| ImPPO[13] | 0.315 | 0.685 | 0.46 | 1 | 0.04705 | Not reported | Not reported | Not reported |
| C18BQAPPO-3[14] | 0.26 | 0.74 | 0.351 | 1 | 0.08517 | Not reported | Not reported | Not reported |
| QPPO-4[14] | 0.36 | 0.64 | 0.563 | 1 | 0.0544 | Not reported | Not reported | Not reported |
| BQAPPO-0.23[15] | 0.23 | 0.77 | 0.299 | 1 | 0.0985 | 2.1-2.3 | Not reported | Fully hydrated; |
| TQAPPO-0.17[15] | 0.17 | 0.83 | 0.205 | 1 | 0.1188 | | | |
| IMPES-0.30[16] | 0.03 | 0.1 | 0.3 | 1 | 0.057479 | Not reported | Not reported | Not reported |
| ImPES-0.35[16] | 0.035 | 0.1 | 0.35 | 1 | 0.072055 | Not reported | Not reported | Not reported |
| ImPES-0.40[16] | 0.04 | 0.1 | 0.4 | 1 | 0.08937 | Not reported | Not reported | Not reported |
| ImPES-0.45[16] | 0.045 | 0.1 | 0.4 | 1 | 0.112712 | Not reported | Not reported | Not reported |
| PMP-TMA-4[17] | 0.0042 | 0.1 | 0.042 | 1 | 0.017943 | Not reported | Not reported | Not reported |
| PMP-TMA-20[17] | 0.02 | 0.1 | 0.2 | 1 | 0.05287 | Not reported | Not reported | Not reported |
| PMP-TMA-32[17] | 0.032 | 0.1 | 0.32 | 1 | 0.069163 | Not reported | Not reported | Not reported |

| | | | | | | | | |
|---|---|---|---|---|---|---|---|---|
| PMP-TMA-41[17] | 0.041 | 0.1 | 0.41 | 1 | 0.075504 | Not reported | Not reported | Not reported |
| PAES-Im-22[18] | 0.22 | 0.78 | 0.282 | 1 | 0.0253 | Not reported | Not reported | Not reported |
| PAES-Im-28[18] | 0.28 | 0.72 | 0.389 | 1 | 0.0309 | Not reported | Not reported | Not reported |
| PAES-Im-34[18] | 0.34 | 0.66 | 0.515 | 1 | 0.0359 | Not reported | Not reported | Not reported |
| PAES-QA[18] | 0.25 | 0.75 | 0.333 | 1 | 0.0361 | Not reported | Not reported | Not reported |
| TA-1C-1.22[19] | 0.393 | 0.607 | 0.647 | 1 | 0.107487 | Not reported | Not reported | Not reported |
| TA-7C-1.25[19] | 0.473 | 0.527 | 0.898 | 1 | 0.109529 | Not reported | Not reported | Not reported |
| TA-10C-1.24[19] | 0.52 | 0.48 | 1.083 | 1 | 0.131047 | Not reported | Not reported | Not reported |
| TA-14C-1.21[19] | 0.543 | 0.457 | 1.188 | 1 | 0.142984 | Not reported | Not reported | Not reported |
| QA-10C-1.27[19] | 0.487 | 0.513 | 0.949 | 1 | 0.042932 | Not reported | Not reported | Not reported |
| PAES-Q-12[20] | 0.12 | 0.88 | 0.136 | 1 | 0.054 | Not reported | Not reported | Not reported |
| PAES-Q-16[20] | 0.16 | 0.84 | 0.19 | 1 | 0.071 | Not rep | Not reported | Not reported |

| | | | | | | | | |
|---|---|---|---|---|---|---|---|---|
| | | | | | | ort ed | | |
| PAES-Q-20[20] | 0.2 | 0.8 | 0.25 | 1 | 0.082 | Not rep ort ed | Not reported | Not reported |
| QAPPO-30[21] | 0.3 | 0.7 | 0.429 | 1 | 0.027052 | Not rep ort ed | Not reported | Not reported |
| QAPPO-40[21] | 0.4 | 0.6 | 0.667 | 1 | 0.049509 | Not rep ort ed | Not reported | Not reported |
| SEBS-TMA[22] | 0.26 | 0.74 | 0.351 | 1 | 0.102 | 1-1.5 | In-plane EIS using two-point probe | Fully hydrated; membranes soaked in deassed DI water |
| SEBS-TMHA[22] | 0.26 | 0.74 | 0.351 | 1 | 0.063 | | | |
| QPEN-0.4[23] | 0.4 | 0.6 | 0.667 | 1 | 0.031967 | Not rep ort ed | Not reported | Not reported |
| QPEN-0.5[23] | 0.49 | 0.51 | 0.961 | 1 | 0.043443 | Not rep ort ed | Not reported | Not reported |
| QPEN-0.6[23] | 0.61 | 0.39 | 1.564 | 1 | 0.064098 | Not rep ort ed | Not reported | Not reported |
| QPEN-0.8[23] | 0.81 | 0.19 | 4.263 | 1 | 0.098197 | Not rep ort ed | Not reported | Not reported |
| QPEN-1.0[23] | 0.5 | 0.5 | 1 | 1 | 0.118525 | Not rep ort ed | Not reported | Not reported |
| ImPESN-9-22[24] | 0.287 | 0.713 | 0.403 | 0 | 0.089 | Not rep ort ed | Not reported | Not reported |
| ImPESN-19-22[24] | 0.463 | 0.537 | 0.862 | 1 | 0.114523 | Not rep | Not reported | Not reported |

| | | | | | | | | |
|---|---|---|---|---|---|---|---|---|
| | | | | | | ort ed | | |
| ImPESN-30-22[24] | 0.574 | 0.426 | 1.347 | 1 | 0.1467 | Not reported | Not reported | Not reported |
| M8.5-GOH[25] | 0.085 | 0.915 | 0.0929 | 1 | 0.01301 | Not reported | Not reported | Not reported |
| M12.5-GOH[25] | 0.125 | 0.875 | 0.143 | 1 | 0.01619 | Not reported | Not reported | Not reported |
| M16.0-GOH[25] | 0.16 | 0.84 | 0.19 | 1 | 0.02501 | Not reported | Not reported | Not reported |
| x-PP-TMA-20[26] | 0.2 | 0.8 | 0.25 | 1 | 0.050785 | Not reported | Not reported | Not reported |
| x-PP-DMHDA-20[26] | 0.2 | 0.8 | 0.25 | 1 | 0.052832 | Not reported | Not reported | Not reported |
| PP-TMA-20[26] | 0.2 | 0.8 | 0.25 | 1 | 0.053552 | Not reported | Not reported | Not reported |
| PP-DMHDA-20[26] | 0.2 | 0.8 | 0.25 | 1 | 0.056818 | Not reported | Not reported | Not reported |
| 53% GQ-PEEK[27] | 0.53 | 0.47 | 1.128 | 1 | 0.023937 | Not reported | Not reported | Not reported |
| QPAES-X8Y8 (DCM 3.17)[28] | 0.5 | 0.5 | 1 | 1 | 0.0758 | Not reported | Not reported | Not reported |
| QPAES-X8Y8 (DCM 1.70)[28] | 0.5 | 0.5 | 1 | 1 | 0.0271 | Not reported | Not reported | Not reported |

| | | | | | | | | |
|---|---|---|---|---|---|---|---|---|
| QPAES-X16Y8 (DCM 2.47)[28] | 0.333 | 0.667 | 0.499 | 1 | 0.0515 | Not reported | Not reported | Not reported |
| QPAES-X16Y8 (DCM 1.99)[28] | 0.333 | 0.667 | 0.499 | 1 | 0.0442 | Not reported | Not reported | Not reported |
| QPAES-X20Y8 (DCM 3.69)[28] | 0.286 | 0.714 | 0.401 | 1 | 0.0695 | Not reported | Not reported | Not reported |
| QPAES-X16Y10 (DCM 0.91)[28] | 0.385 | 0.615 | 0.626 | 1 | 0.0121 | Not reported | Not reported | Not reported |
| QPAES-X16Y10 (DCM 2.35)[28] | 0.385 | 0.615 | 0.626 | 1 | 0.0378 | Not reported | Not reported | Not reported |
| QPAES-X16Y10 (DCM 2.64)[28] | 0.385 | 0.615 | 0.626 | 1 | 0.0545 | Not reported | Not reported | Not reported |
| QPAES-X20Y10 (DCM 2.73)[28] | 0.333 | 0.667 | 0.499 | 1 | 0.0586 | Not reported | Not reported | Not reported |
| QPAES-X20Y18 (DCM 2.00)[28] | 0.474 | 0.526 | 0.901 | 1 | 0.0357 | Not reported | Not reported | Not reported |
| QPAES-X20Y18 (DCM 2.57)[28] | 0.474 | 0.526 | 0.901 | 1 | 0.0641 | Not reported | Not reported | Not reported |
| PAES-Q-90[29] | 0.62 | 0.38 | 1.632 | 1 | 0.093 | Not reported | Not reported | Not reported |
| PAES-Q-80[29] | 0.46 | 0.54 | 0.852 | 1 | 0.0679 | Not reported | Not reported | Not reported |
| PAES-Q-75[29] | 0.33 | 0.67 | 0.493 | 1 | 0.0473 | Not rep | Not reported | Not reported |

| | | | | | | | | |
|---|---|---|---|---|---|---|---|---|
| | | | | | | ort ed | | |
| MPAES-Q-1[29] | 0.06 | 0.94 | 0.064 | 1 | 0.0456 | Not rep ort ed | Not reported | Not reported |
| MPAES-Q-2[29] | 0.36 | 0.64 | 0.563 | 1 | 0.0281 | Not rep ort ed | Not reported | Not reported |
| QPE-TMA-X5Y9[30] | 0.643 | 0.357 | 1.801 | 1 | 0.06267 | Not rep ort ed | Not reported | Not reported |
| QPE-TMA-X5Y3[30] | 0.375 | 0.625 | 0.6 | 1 | 0.01414 | Not rep ort ed | Not reported | Not reported |
| QPE-MIm-X5Y9[30] | 0.643 | 0.357 | 1.801 | 1 | 0.02079 | Not rep ort ed | Not reported | Not reported |
| QPE-DMIm-X5Y3[30] | 0.375 | 0.625 | 0.6 | 1 | 0.02036 | Not rep ort ed | Not reported | Not reported |
| QPE-BDMA-X5Y3[30] | 0.375 | 0.625 | 0.6 | 1 | 0.00709 | Not rep ort ed | Not reported | Not reported |
| QPE-PYR-X5Y3[30] | 0.375 | 0.625 | 0.6 | 1 | 0.00126 | Not rep ort ed | Not reported | Not reported |
| QBMPAE-a (DBM 0.8)[31] | 0.8 | 0.2 | 4 | 1 | 0.0121 | Not rep ort ed | Not reported | Not reported |
| QBMPAE-a (DBM 1.21)[31] | 0.174 | 0.826 | 0.211 | 1 | 0.0234 | Not rep ort ed | Not reported | Not reported |
| QBMPAE-b (DBM 0.77)[31] | 0.77 | 0.23 | 3.348 | 1 | 0.014 | Not rep ort ed | Not reported | Not reported |

| | | | | | | | | |
|---|---|---|---|---|---|---|---|---|
| QBMPAE-b (DBM 1.36)[31] | 0.265 | 0.735 | 0.361 | 1 | 0.0398 | Not reported | Not reported | Not reported |
| QBMPAE-c (DBM 0.8)[31] | 0.8 | 0.2 | 4 | 1 | 0.0055 | Not reported | Not reported | Not reported |
| QBMPAE-c (DBM 1.42)[31] | 0.296 | 0.704 | 0.42 | 1 | 0.0254 | Not reported | Not reported | Not reported |
| QBMPAE-d (DBM 0.83)[31] | 0.83 | 0.17 | 4.882 | 1 | 0.0215 | Not reported | Not reported | Not reported |
| QBMPAE-d (DBM 1.38)[31] | 0.275 | 0.725 | 0.379 | 1 | 0.0466 | Not reported | Not reported | Not reported |
| BTMA40[32] | 0.4 | 0.6 | 0.667 | 1 | 0.044 | Not reported | Not reported | Not reported |
| S60NC6[32] | 0.6 | 0.4 | 1.5 | 1 | 0.0944 | Not reported | Not reported | Not reported |
| D30NC6NC6[32] | 0.3 | 0.7 | 0.429 | 1 | 0.156 | Not reported | Not reported | Not reported |
| T20NC6NC5N[32] | 0.2 | 0.8 | 0.25 | 1 | 0.1752 | Not reported | Not reported | Not reported |
| AI-PES-2[33] | 0.5 | 0.5 | 1 | 1 | 0.075 | Not reported | Not reported | Not reported |
| AI-PES-6[33] | 0.5 | 0.5 | 1 | 1 | 0.11 | Not reported | Not reported | Not reported |
| AI-PES-12[33] | 0.5 | 0.5 | 1 | 1 | 0.14 | Not rep | Not reported | Not reported |

| | | | | | | | | |
|---|---|---|---|---|---|---|---|---|
| | | | | | | ort ed | | |
| AI-PES-16[33] | 0.5 | 0.5 | 1 | 1 | 0.12 | Not reported | Not reported | Not reported |
| PSf-PDApip1[34] | 0.318 | 0.682 | 0.466 | 1 | 0.025 | Not reported | Not reported | Not reported |
| PSf-PDApip2[34] | 0.409 | 0.591 | 0.692 | 1 | 0.06 | Not reported | Not reported | Not reported |
| PSf-PDApip3[34] | 0.496 | 0.504 | 0.984 | 1 | 0.079 | Not reported | Not reported | Not reported |
| PSf-PDApip4[34] | 0.618 | 0.382 | 1.618 | 1 | 0.102 | Not reported | Not reported | Not reported |
| QAPIB[35] | 0.42 | 0.58 | 0.724 | 1 | 0.09388 | Not reported | Not reported | Not reported |
| PyrPIB[35] | 0.84 | 0.16 | 5.25 | 1 | 0.04926 | Not reported | Not reported | Not reported |
| PipPIB[35] | 0.6 | 0.4 | 1.5 | 1 | 0.04319 | Not reported | Not reported | Not reported |
| PAP-TP-75[36] | 0.75 | 0.25 | 3 | 1 | 0.1442 | Not reported | Not reported | Not reported |
| PAP-TP-80[36] | 0.8 | 0.2 | 4 | 1 | 0.1567 | Not reported | Not reported | Not reported |
| PAP-TP-85[36] | 0.85 | 0.15 | 5.667 | 1 | 0.16902 | Not reported | Not reported | Not reported |

| | | | | | | | | |
|---|---|---|---|---|---|---|---|---|
| PAP-BP-60[36] | 0.6 | 0.4 | 1.5 | 1 | 0.12572 | Not reported | Not reported | Not reported |
| PAP-BP-70[36] | 0.7 | 0.3 | 2.333 | 1 | 0.1442 | Not reported | Not reported | Not reported |
| ABA-QA-1[37] | 0.502 | 0.498 | 1.007 | 1 | 0.04743 | Not reported | Not reported | Not reported |
| ABA-QA-2[37] | 0.567 | 0.433 | 1.309 | 1 | 0.07126 | Not reported | Not reported | Not reported |
| ABA-QA-3[37] | 0.725 | 0.275 | 2.636 | 1 | 0.105561 | Not reported | Not reported | Not reported |
| BPi[38] | 0.3 | 0.7 | 0.429 | 1 | 0.03982 | Not reported | Not reported | Not reported |
| SCPi[38] | 0.3 | 0.7 | 0.429 | 1 | 0.05282 | Not reported | Not reported | Not reported |
| LSCPi[38] | 0.3 | 0.7 | 0.429 | 1 | 0.06485 | Not reported | Not reported | Not reported |
| CQA-20[39] | 0.2 | 0.8 | 0.16 | 1 | 0.0114 | Not reported | Not reported | Not reported |
| CQA-30[39] | 0.3 | 0.7 | 0.429 | 1 | 0.041 | Not reported | Not reported | Not reported |
| LSCQA-14[39] | 0.14 | 0.86 | 0.163 | 1 | 0.0386 | Not reported | Not reported | Not reported |
| LSCQA-20[39] | 0.2 | 0.8 | 0.16 | 1 | 0.0534 | Not rep | Not reported | Not reported |

| | | | | | | | | |
|---|---|---|---|---|---|---|---|---|
| | | | | | | ort ed | | |
| LSCQA-30[39] | 0.3 | 0.7 | 0.429 | 1 | 0.089 | Not reported | Not reported | Not reported |
| SCCQA4-20[39] | 0.2 | 0.8 | 0.16 | 1 | 0.0098 | Not reported | Not reported | Not reported |
| SCCQA4-30[39] | 0.3 | 0.7 | 0.429 | 1 | 0.0225 | Not reported | Not reported | Not reported |
| SCCQA4-40[39] | 0.4 | 0.6 | 0.667 | 1 | 0.0322 | Not reported | Not reported | Not reported |
| SCCQA18-20[39] | 0.2 | 0.8 | 0.16 | 1 | 0.0213 | Not reported | Not reported | Not reported |
| SCCQA18-30[39] | 0.3 | 0.7 | 0.429 | 1 | 0.0386 | Not reported | Not reported | Not reported |
| OH-QAPPT[40] | 0.5 | 0.5 | 1 | 1 | 0.137 | Not reported | Not reported | Not reported |
| PTP[41] | 0.5 | 0.5 | 1 | 0 | 0.12085 | Not reported | Not reported | Not reported |
| PDTP-25[41] | 0.75 | 0.25 | 3 | 0 | 0.16496 | Not reported | Not reported | Not reported |
| PDTP-50[41] | 0.5 | 0.5 | 1 | 1 | 0.1578 | Not reported | Not reported | Not reported |
| PDPF-TMA[42] | 0.89 | 0.11 | 8.091 | 1 | 0.107 | Not reported | Not reported | Not reported |

| | | | | | | | | |
|---|---|---|---|---|---|---|---|---|
| PDPF-Pip[42] | 0.89 | 0.11 | 8.091 | 1 | 0.075 | Not reported | Not reported | Not reported |
| PDPF-Qui[42] | 0.89 | 0.11 | 8.091 | 1 | 0.1 | Not reported | Not reported | Not reported |
| PES-PPH-Pi(OH)-100[43] | 0.996 | 0.004 | 249 | 1 | 0.056 | Not reported | Not reported | Not reported |
| PES-PPH-Pi(OH)-80[43] | 0.797 | 0.203 | 3.926 | 1 | 0.053 | Not reported | Not reported | Not reported |
| PES-PPH-Mor(OH)-100[43] | 0.978 | 0.022 | 44.455 | 1 | 0.048 | Not reported | Not reported | Not reported |
| PES-PPH-Mor(OH)-80[43] | 0.752 | 0.248 | 3.032 | 1 | 0.04 | Not reported | Not reported | Not reported |
| PES-PPH-Py(OH)-100[43] | 0.974 | 0.026 | 37.462 | 1 | 0.059 | Not reported | Not reported | Not reported |
| PES-PPH-Py(OH)-80[43] | 0.785 | 0.215 | 3.651 | 1 | 0.055 | Not reported | Not reported | Not reported |
| PPO-SDSU-20[44] | 0.2 | 0.8 | 0.25 | 1 | 0.0408 | Not reported | Not reported | Not reported |
| PPO-SDSU-27[44] | 0.27 | 0.73 | 0.37 | 1 | 0.05011 | Not reported | Not reported | Not reported |
| PPO-SDSU-36[44] | 0.36 | 0.64 | 0.563 | 1 | 0.07691 | Not reported | Not reported | Not reported |
| PPO-DDSU-20[44] | 0.2 | 0.8 | 0.25 | 1 | 0.04817 | Not rep | Not reported | Not reported |

| | | | | | | | | |
|---|---|---|---|---|---|---|---|---|
| | | | | | | ort ed | | |
| PPO-DDSU-27[44] | 0.27 | 0.73 | 0.37 | 1 | 0.07028 | Not reported | Not reported | Not reported |
| QAPTBHI[45] | 0.5 | 0.5 | 1 | 1 | 0.0971 | Not reported | Not reported | Not reported |
| QAPTDBHI-20[45] | 0.8 | 0.2 | 4 | 0 | 0.11556 | Not reported | Not reported | Not reported |
| QAPTDBHI-30[45] | 0.7 | 0.3 | 2.333 | 1 | 0.13594 | Not reported | Not reported | Not reported |
| QAPTDBHI-40[45] | 0.6 | 0.4 | 1.5 | 1 | 0.15159 | Not reported | Not reported | Not reported |
| P5Ph-1.8[46] | 0.598 | 0.402 | 1.488 | 1 | 0.06508 | Not reported | Not reported | Not reported |
| P6Ph-2.2[46] | 0.739 | 0.261 | 2.831 | 1 | 0.09541 | Not reported | Not reported | Not reported |
| P5Ph-2.3[46] | 0.746 | 0.254 | 2.937 | 1 | 0.10068 | Not reported | Not reported | Not reported |
| P5Me-2.2[46] | 0.678 | 0.332 | 2.106 | 1 | 0.09522 | Not reported | Not reported | Not reported |
| P6Me-2.0[46] | 0.645 | 0.355 | 1.817 | 1 | 0.10463 | Not reported | Not reported | Not reported |
| PPO-7Q-0.8[47] | 0.136 | 0.864 | 0.157 | 1 | 0.00115 | Not reported | Not reported | Not reported |

| | | | | | | | | |
|---|---|---|---|---|---|---|---|---|
| PPO-7Q-1.2[47] | 0.217 | 0.783 | 0.277 | 1 | 0.03914 | Not reported | Not reported | Not reported |
| PPO-7Q-1.3[47] | 0.24 | 0.76 | 0.316 | 1 | 0.05101 | Not reported | Not reported | Not reported |
| PPO-7Q-1.5[47] | 0.286 | 0.714 | 0.401 | 1 | 0.06337 | Not reported | Not reported | Not reported |
| PPO-7Q-1.8[47] | 0.363 | 0.637 | 0.57 | 1 | 0.0829 | Not reported | Not reported | Not reported |
| PPO-1Q-0.8[47] | 0.126 | 0.874 | 0.144 | 1 | 0.00793 | Not reported | Not reported | Not reported |
| PPO-1Q-1.5[47] | 0.247 | 0.753 | 0.328 | 1 | 0.01242 | Not reported | Not reported | Not reported |
| PTPipQ100[48] | 0.5 | 0.5 | 1 | 1 | 0.13332 | Not reported | Not reported | Not reported |
| PTPipQ83[48] | 0.83 | 0.17 | 4.882 | 0 | 0.13707 | Not reported | Not reported | Not reported |
| PpTASU[48] | 0.5 | 0.5 | 1 | 1 | 0.15706 | Not reported | Not reported | Not reported |
| PpTDMP[48] | 0.5 | 0.5 | 1 | 0 | 0.17819 | Not reported | Not reported | Not reported |
| PmTASU[48] | 0.5 | 0.5 | 1 | 0 | 0.14711 | Not reported | Not reported | Not reported |
| PmTDMP[48] | 0.5 | 0.5 | 1 | 0 | 0.16904 | Not rep | Not reported | Not reported |

| | | | | | | | | |
|---|---|---|---|---|---|---|---|---|
| | | | | | | ort ed | | |
| PPO-Pip[49] | 0.34 | 0.66 | 0.515 | 0 | 0.05625 | Not rep ort ed | Not reported | Not reported |
| PPO-OPip[49] | 0.34 | 0.66 | 0.515 | 1 | 0.06349 | Not rep ort ed | Not reported | Not reported |
| PPO-PipOH[49] | 0.34 | 0.66 | 0.515 | 1 | 0.06541 | Not rep ort ed | Not reported | Not reported |
| F20C9N[50] | 0.2 | 0.8 | 0.25 | 1 | 0.06976 | Not rep ort ed | Not reported | Not reported |
| H22C9N[50] | 0.22 | 0.78 | 0.282 | 1 | 0.067835 | Not rep ort ed | Not reported | Not reported |
| PAEK-HQACz-0.5[51] | 0.5 | 0.5 | 1 | 1 | 0.05063 | Not rep ort ed | Not reported | Not reported |
| PAEK-HQACz-0.6[51] | 0.6 | 0.4 | 1.5 | 1 | 0.07172 | Not rep ort ed | Not reported | Not reported |
| PAEK-HQACz-0.7[51] | 0.7 | 0.3 | 2.333 | 1 | 0.09873 | Not rep ort ed | Not reported | Not reported |

**Supporting References**

(1) Kim, S.; Yang, S.; Kim, D. Poly(arylene ether ketone) with pendant pyridinium groups for alkaline fuel cell membranes. *International Journal of Hydrogen Energy* **2017**, *42* (17), 12496-12506. DOI: https://doi.org/10.1016/j.ijhydene.2017.03.187.

(2) Kim, D. J.; Lee, B.-N.; Nam, S. Y. Synthesis and characterization of PEEK containing imidazole for anion exchange membrane fuel cell. *International Journal of Hydrogen Energy* **2017**, *42* (37), 23759-23767. DOI: https://doi.org/10.1016/j.ijhydene.2017.02.199.
(3) Irfan, M.; Bakangura, E.; Afsar, N. U.; Hossain, M. M.; Ran, J.; Xu, T. Preparation and performance evaluation of novel alkaline stable anion exchange membranes. *Journal of Power Sources* **2017**, *355*, 171-180. DOI: https://doi.org/10.1016/j.jpowsour.2017.03.146.
(4) Lin, C.; Huang, X.; Guo, D.; Zhang, Q.-Q.; Zhu, A.; Ling, Y.; Liu, Q. L. Side-chain-type anion exchange membranes bearing pendant quaternary ammonium groups via flexible spacer for fuel cells. *J. Mater. Chem. A* **2016**, *4*. DOI: 10.1039/C6TA05090E.
(5) Zhang, X.; Li, S.; Chen, P.; Fang, J.; Shi, Q.; Weng, Q.; Luo, X.; Chen, X.; An, Z. Imidazolium functionalized block copolymer anion exchange membrane with enhanced hydroxide conductivity and alkaline stability via tailoring side chains. *International Journal of Hydrogen Energy* **2018**, *43* (7), 3716-3730. DOI: https://doi.org/10.1016/j.ijhydene.2018.01.017.
(6) Lu, D.; Li, D.; Wen, L.; Xue, L. Effects of non-planar hydrophobic cyclohexylidene moiety on the structure and stability of poly(arylene ether sulfone)s based anion exchange membranes. *Journal of Membrane Science* **2017**, *533*, 210-219. DOI: https://doi.org/10.1016/j.memsci.2017.03.011.
(7) Lin, C. X.; Zhuo, Y. Z.; Lai, A. N.; Zhang, Q. G.; Zhu, A. M.; Ye, M. L.; Liu, Q. L. Side-chain-type anion exchange membranes bearing pendent imidazolium-functionalized poly(phenylene oxide) for fuel cells. *Journal of Membrane Science* **2016**, *513*, 206-216. DOI: https://doi.org/10.1016/j.memsci.2016.04.054.
(8) Guo, D.; Lin, C. X.; Hu, E. N.; Shi, L.; Soyekwo, F.; Zhang, Q. G.; Zhu, A. M.; Liu, Q. L. Clustered multi-imidazolium side chains functionalized alkaline anion exchange membranes for fuel cells. *Journal of Membrane Science* **2017**, *541*, 214-223. DOI: https://doi.org/10.1016/j.memsci.2017.07.007.
(9) Lee, J. Y.; Lim, D.-H.; Chae, J. E.; Choi, J.; Kim, B. H.; Lee, S. Y.; Yoon, C. W.; Nam, S. Y.; Jang, J. H.; Henkensmeier, D.; et al. Base tolerant polybenzimidazolium hydroxide membranes for solid alkaline-exchange membrane fuel cells. *Journal of Membrane Science* **2016**, *514*, 398-406. DOI: https://doi.org/10.1016/j.memsci.2016.05.012.
(10) Kwon, S.; Rao, A.; Kim, T.-H. Anion exchange membranes based on terminally crosslinked methyl morpholinium-functionalized poly(arylene ether sulfone)s. *Journal of Power Sources* **2017**, *375*. DOI: 10.1016/j.jpowsour.2017.06.047.
(11) Lai, A. N.; Zhou, K.; Zhuo, Y. Z.; Zhang, Q. G.; Zhu, A. M.; Ye, M. L.; Liu, Q. L. Anion exchange membranes based on carbazole-containing polyolefin for direct methanol fuel cells. *Journal of Membrane Science* **2016**, *497*, 99-107. DOI: https://doi.org/10.1016/j.memsci.2015.08.069.
(12) Fang, J.; Lyu, M.; Wang, X.; Wu, Y.; Zhao, J. Synthesis and performance of novel anion exchange membranes based on imidazolium ionic liquids for alkaline fuel cell applications. *Journal of Power Sources* **2015**, *284*, 517-523. DOI: https://doi.org/10.1016/j.jpowsour.2015.03.065.
(13) Yang, Q.; Lin, C. X.; Liu, F. H.; Li, L.; Zhang, Q. G.; Zhu, A. M.; Liu, Q. L. Poly (2,6-dimethyl-1,4-phenylene oxide)/ionic liquid functionalized graphene oxide anion exchange membranes for fuel cells. *Journal of Membrane Science* **2018**, *552*, 367-376. DOI: https://doi.org/10.1016/j.memsci.2018.02.036.
(14) He, Y.; Si, J.; Wu, L.; Chen, S.; Zhu, Y.; Pan, J.; Ge, X.; Yang, Z.; Xu, T. Dual-cation comb-shaped anion exchange membranes: Structure, morphology and properties. *Journal of Membrane Science* **2016**, *515*, 189-195. DOI: https://doi.org/10.1016/j.memsci.2016.05.058.
(15) He, Y.; Pan, J.; Wu, L.; Zhu, Y.; Ge, X.; Ran, J.; Yang, Z.; Xu, T. A Novel Methodology to Synthesize Highly Conductive Anion Exchange Membranes. *Scientific Reports* **2015**, *5* (1), 13417. DOI: 10.1038/srep13417.
(16) Guo, D.; Lai, A. N.; Lin, C. X.; Zhang, Q. G.; Zhu, A. M.; Liu, Q. L. Imidazolium-Functionalized Poly(arylene ether sulfone) Anion-Exchange Membranes Densely Grafted with Flexible Side Chains for Fuel Cells. *ACS Applied Materials & Interfaces* **2016**, *8* (38), 25279-25288. DOI: 10.1021/acsami.6b07711.
(17) Zhang, M.; Shan, C.; Liu, L.; Liao, J.; Chen, Q.; Zhu, M.; Wang, Y.; An, L.; Li, N. Facilitating Anion Transport in Polyolefin-Based Anion Exchange Membranes via Bulky Side Chains. *ACS Applied Materials & Interfaces* **2016**, *8* (35), 23321-23330. DOI: 10.1021/acsami.6b06426.
(18) Wang, C.; Xu, C.; Shen, B.; Zhao, X.; Li, J. Stable poly(arylene ether sulfone)s anion exchange membranes containing imidazolium cations on pendant phenyl rings. *Electrochimica Acta* **2016**, *190*, 1057-1065. DOI: https://doi.org/10.1016/j.electacta.2015.12.181.

(19) Ge, Q.; Ran, J.; Miao, J.; Yang, Z.; Xu, T. Click Chemistry Finds Its Way in Constructing an Ionic Highway in Anion-Exchange Membrane. *ACS Applied Materials & Interfaces* **2015**, *7* (51), 28545-28553. DOI: 10.1021/acsami.5b09920.
(20) Wang, C.; Shen, B.; Xu, C.; Zhao, X.; Li, J. Side-chain-type poly(arylene ether sulfone)s containing multiple quaternary ammonium groups as anion exchange membranes. *Journal of Membrane Science* **2015**, *492*, 281-288. DOI: https://doi.org/10.1016/j.memsci.2015.05.060.
(21) Pan, J.; Zhu, L.; Han, J.; Hickner, M. A. Mechanically Tough and Chemically Stable Anion Exchange Membranes from Rigid-Flexible Semi-Interpenetrating Networks. *Chemistry of Materials* **2015**, *27* (19), 6689-6698. DOI: 10.1021/acs.chemmater.5b02557.
(22) Mohanty, A. D.; Ryu, C. Y.; Kim, Y. S.; Bae, C. Stable Elastomeric Anion Exchange Membranes Based on Quaternary Ammonium-Tethered Polystyrene-b-poly(ethylene-co-butylene)-b-polystyrene Triblock Copolymers. *Macromolecules* **2015**, *48* (19), 7085-7095. DOI: 10.1021/acs.macromol.5b01382.
(23) Lai, A. N.; Wang, L. S.; Lin, C. X.; Zhuo, Y. Z.; Zhang, Q. G.; Zhu, A. M.; Liu, Q. L. Benzylmethyl-containing poly(arylene ether nitrile) as anion exchange membranes for alkaline fuel cells. *Journal of Membrane Science* **2015**, *481*, 9-18. DOI: https://doi.org/10.1016/j.memsci.2015.02.013.
(24) Lai, A. N.; Wang, L. S.; Lin, C. X.; Zhuo, Y. Z.; Zhang, Q. G.; Zhu, A. M.; Liu, Q. L. Phenolphthalein-based Poly(arylene ether sulfone nitrile)s Multiblock Copolymers As Anion Exchange Membranes for Alkaline Fuel Cells. *ACS Applied Materials & Interfaces* **2015**, *7* (15), 8284-8292. DOI: 10.1021/acsami.5b01475.
(25) Sherazi, T. A.; Zahoor, S.; Raza, R.; Shaikh, A. J.; Naqvi, S. A. R.; Abbas, G.; Khan, Y.; Li, S. Guanidine functionalized radiation induced grafted anion-exchange membranes for solid alkaline fuel cells. *International Journal of Hydrogen Energy* **2015**, *40* (1), 786-796. DOI: https://doi.org/10.1016/j.ijhydene.2014.08.086.
(26) Zhang, M.; Liu, J.; Wang, Y.; An, L.; Guiver, M.; Li, N. Highly Stable Anion Exchange Membranes Based on Quaternized Polypropylene. *J. Mater. Chem. A* **2015**, *3*. DOI: 10.1039/C5TA01420D.
(27) Si, J.; Lu, S.; Xu, X.; Peng, S.; Xiu, R.; Xiang, Y. A Gemini Quaternary Ammonium Poly (ether ether ketone) Anion-Exchange Membrane for Alkaline Fuel Cell: Design, Synthesis, and Properties. *ChemSusChem* **2014**, *7* (12), 3389-3395. DOI: https://doi.org/10.1002/cssc.201402664 (acccessed 2025/09/14).
(28) Li, X.; Liu, Q.; Yu, Y.; Meng, Y. Synthesis and properties of multiblock ionomers containing densely functionalized hydrophilic blocks for anion exchange membranes. *Journal of Membrane Science* **2014**, *467*, 1-12. DOI: https://doi.org/10.1016/j.memsci.2014.05.016.
(29) Li, X.; Nie, G.; Tao, J.; Wu, W.; Wang, L.; Liao, S. Assessing the Influence of Side-Chain and Main-Chain Aromatic Benzyltrimethyl Ammonium on Anion Exchange Membranes. *ACS Applied Materials & Interfaces* **2014**, *6* (10), 7585-7595. DOI: 10.1021/am500915w.
(30) Miyake, J.; Fukasawa, K.; Watanabe, M.; Miyatake, K. Effect of ammonium groups on the properties and alkaline stability of poly(arylene ether)-based anion exchange membranes. *Journal of Polymer Science Part A: Polymer Chemistry* **2014**, *52* (3), 383-389. DOI: https://doi.org/10.1002/pola.27011 (acccessed 2025/09/14).
(31) Li, X.; Cheng, S.; Wang, L.; Long, Q.; Tao, J.; Nie, G.; Liao, S. Anion exchange membranes by bromination of benzylmethyl-containing poly(arylene ether)s for alkaline membrane fuel cells. *RSC Advances* **2014**, *4* (56), 29682-29693, 10.1039/C4RA00833B. DOI: 10.1039/C4RA00833B.
(32) Zhu, L.; Pan, J.; Wang, Y.; Han, J.; Zhuang, L.; Hickner, M. A. Multication Side Chain Anion Exchange Membranes. *Macromolecules* **2016**, *49* (3), 815-824. DOI: 10.1021/acs.macromol.5b02671.
(33) Rao, A. H. N.; Nam, S.; Kim, T.-H. Comb-shaped alkyl imidazolium-functionalized poly(arylene ether sulfone)s as high performance anion-exchange membranes. *Journal of Materials Chemistry A* **2015**, *3* (16), 8571-8580, 10.1039/C5TA01123J. DOI: 10.1039/C5TA01123J.
(34) Strasser, D.; Graziano, B.; Knauss, D. Base stable poly(diallylpiperidinium hydroxide) multiblock copolymers for anion exchange membranes. *J. Mater. Chem. A* **2017**, *5*. DOI: 10.1039/C7TA00905D.
(35) Zhang, S.; Zhu, X.; Jin, C. Development of a high-performance anion exchange membrane using poly(isatin biphenylene) with flexible heterocyclic quaternary ammonium cations for alkaline fuel cells. *Journal of Materials Chemistry A* **2019**, *7* (12), 6883-6893, 10.1039/C8TA11291F. DOI: 10.1039/C8TA11291F.
(36) Wang, J.; Zhao, Y.; Setzler, B. P.; Rojas-Carbonell, S.; Ben Yehuda, C.; Amel, A.; Page, M.; Wang, L.; Hu, K.; Shi, L.; et al. Poly(aryl piperidinium) membranes and ionomers for hydroxide exchange membrane fuel cells. *Nature Energy* **2019**, *4* (5), 392-398. DOI: 10.1038/s41560-019-0372-8.

(37) Lin, C. X.; Wang, X. Q.; Li, L.; Liu, F. H.; Zhang, Q. G.; Zhu, A. M.; Liu, Q. L. Triblock copolymer anion exchange membranes bearing alkyl-tethered cycloaliphatic quaternary ammonium-head-groups for fuel cells. *Journal of Power Sources* **2017**, *365*, 282-292. DOI: https://doi.org/10.1016/j.jpowsour.2017.08.100.
(38) Chu, X.; Shi, Y.; Liu, L.; Huang, Y.; Li, N. Piperidinium-functionalized anion exchange membranes and their application in alkaline fuel cells and water electrolysis. *Journal of Materials Chemistry A* **2019**, *7* (13), 7717-7727, 10.1039/C9TA01167F. DOI: 10.1039/C9TA01167F.
(39) Liu, L.; Chu, X.; Liao, J.; Huang, Y.; Li, Y.; Ge, Z.; Hickner, M. A.; Li, N. Tuning the properties of poly(2,6-dimethyl-1,4-phenylene oxide) anion exchange membranes and their performance in H2/O2 fuel cells. *Energy & Environmental Science* **2018**, *11* (2), 435-446, 10.1039/C7EE02468A. DOI: 10.1039/C7EE02468A.
(40) Peng, H.; Li, Q.; Hu, M.; Xiao, L.; Lu, J.; Zhuang, L. Alkaline polymer electrolyte fuel cells stably working at 80 °C. *Journal of Power Sources* **2018**, *390*, 165-167. DOI: https://doi.org/10.1016/j.jpowsour.2018.04.047.
(41) Chen, N.; Hu, C.; Wang, H. H.; Kim, S. P.; Kim, H. M.; Lee, W. H.; Bae, J. Y.; Park, J. H.; Lee, Y. M. Poly(Alkyl-Terphenyl Piperidinium) Ionomers and Membranes with an Outstanding Alkaline-Membrane Fuel-Cell Performance of 2.58 W cm(-2). *Angew Chem Int Ed Engl* **2021**, *60* (14), 7710-7718. DOI: 10.1002/anie.202013395 From NLM.
(42) Allushi, A.; Pham, T. H.; Olsson, J. S.; Jannasch, P. Ether-free polyfluorenes tethered with quinuclidinium cations as hydroxide exchange membranes. *Journal of Materials Chemistry A* **2019**, *7* (47), 27164-27174, 10.1039/C9TA09213G. DOI: 10.1039/C9TA09213G.
(43) Liu, R.; Wang, J.; Che, X.; Wang, T.; Aili, D.; Li, Q.; Yang, J. Facile synthesis and properties of poly(ether ketone cardo)s bearing heterocycle groups for high temperature polymer electrolyte membrane fuel cells. *Journal of Membrane Science* **2021**, *636*, 119584. DOI: https://doi.org/10.1016/j.memsci.2021.119584.
(44) Xue, J.; Liu, X.; Zhang, J.; Yin, Y.; Guiver, M. D. Poly(phenylene oxide)s incorporating N-spirocyclic quaternary ammonium cation/cation strings for anion exchange membranes. *Journal of Membrane Science* **2020**, *595*, 117507. DOI: https://doi.org/10.1016/j.memsci.2019.117507.
(45) Tian, L.; Ma, W.; Tuo, S.; Wang, F.; Zhu, H. Novel polyaryl isatin polyelectrolytes with flexible monomers for anion exchange membrane fuel cells. *Journal of Membrane Science* **2024**, *690*, 122172. DOI: https://doi.org/10.1016/j.memsci.2023.122172.
(46) Pham, T. H.; Olsson, J. S.; Jannasch, P. Poly(arylene alkylene)s with pendant N-spirocyclic quaternary ammonium cations for anion exchange membranes. *Journal of Materials Chemistry A* **2018**, *6* (34), 16537-16547, 10.1039/C8TA04699A. DOI: 10.1039/C8TA04699A.
(47) Dang, H.-S.; Weiber, E.; Jannasch, P. Poly(phenylene oxide) functionalized with quaternary ammonium groups via flexible alkyl spacers for high-performance anion exchange membranes. *J. Mater. Chem. A* **2015**, *3*. DOI: 10.1039/C5TA00350D.
(48) Pan, D.; Bakvand, P.; Pham, H.; Jannasch, P. Improving poly(arylene piperidinium) anion exchange membranes by monomer design. *Journal of Materials Chemistry A* **2022**, *10*. DOI: 10.1039/D2TA03862E.
